# Gene-nutrient interactions that impact magnesium homeostasis increase risk for neural tube defects in mice exposed to dolutegravir

**DOI:** 10.3389/fcell.2023.1175917

**Published:** 2023-06-12

**Authors:** J. Gelineau-van Waes, M. A. van Waes, J. Hallgren, J. Hulen, M. Bredehoeft, A. E. Ashley-Koch, D. Krupp, S. G. Gregory, H. A. Stessman

**Affiliations:** ^1^ Department of Pharmacology and Neuroscience, School of Medicine, Creighton University, Omaha, NE, United States; ^2^ Independent Researcher, Omaha, NE, United States; ^3^ Duke Molecular Physiology Institute, Duke University Medical Center, Durham, NC, United States; ^4^ Department of Medicine, Duke University Medical Center, Durham, NC, United States; ^5^ Department of Neurosurgery, Duke University Medical Center, Durham, NC, United States

**Keywords:** dolutegravir, antiretroviral therapy, gene-nutrient interactions, neural tube defect, magnesium, hypomagnesemia, maternal diet, FAM111A

## Abstract

In 2018, data from a surveillance study in Botswana evaluating adverse birth outcomes raised concerns that women on antiretroviral therapy (ART) containing dolutegravir (DTG) may be at increased risk for neural tube defects (NTDs). The mechanism of action for DTG involves chelation of Mg^2+^ ions in the active site of the viral integrase. Plasma Mg^2+^ homeostasis is maintained primarily through dietary intake and reabsorption in the kidneys. Inadequate dietary Mg^2+^ intake over several months results in slow depletion of plasma Mg^2+^ and chronic latent hypomagnesemia, a condition prevalent in women of reproductive age worldwide. Mg^2+^ is critical for normal embryonic development and neural tube closure. We hypothesized that DTG therapy might slowly deplete plasma Mg^2+^ and reduce the amount available to the embryo, and that mice with pre-existing hypomagnesemia due to genetic variation and/or dietary Mg^2+^ insufficiency at the time of conception and initiation of DTG treatment would be at increased risk for NTDs. We used two different approaches to test our hypothesis: 1) we selected mouse strains that had inherently different basal plasma Mg^2+^ levels and 2) placed mice on diets with different concentrations of Mg^2+^. Plasma and urine Mg^2+^ were determined prior to timed mating. Pregnant mice were treated daily with vehicle or DTG beginning on the day of conception and embryos examined for NTDs on gestational day 9.5. Plasma DTG was measured for pharmacokinetic analysis. Our results demonstrate that hypomagnesemia prior to conception, due to genetic variation and/or insufficient dietary Mg^2+^ intake, increases the risk for NTDs in mice exposed to DTG. We also analyzed whole-exome sequencing data from inbred mouse strains and identified 9 predicted deleterious missense variants in Fam111a that were unique to the LM/Bc strain. Human FAM111A variants are associated with hypomagnesemia and renal Mg^2+^ wasting. The LM/Bc strain exhibits this same phenotype and was the strain most susceptible to DTG-NTDs. Our results suggest that monitoring plasma Mg^2+^ levels in patients on ART regimens that include DTG, identifying other risk factors that impact Mg^2+^ homeostasis, and correcting deficiencies in this micronutrient might provide an effective strategy for mitigating NTD risk.

## 1 Introduction

Neural tube defects (NTDs) are congenital malformations of the brain and/or spinal cord (anencephaly, spina bifida) that result from failure of the neural tube to close properly during early embryonic development. The etiology of NTDs is multifactorial, involving complex gene-nutrient-environment interactions. Numerous factors that contribute to NTDs are known, including maternal nutritional deficiencies, metabolic disorders (diabetes, obesity), genetic variants, environmental toxins, and pharmaceutical exposures (Finnell et al., 2021; Isaković et al., 2022). It has also been established that the occurrence (Czeizel and Dudas, 1992) or recurrence (MRC Vitamin Study Research Group, 1991; Toriello, 2011; Atta et al., 2016) of NTD-affected pregnancies can be effectively reduced by periconceptional use of folic acid supplements. However, to implement additional strategies for NTD prevention, the continued identification of modifiable or actionable risk factors is critical.

In 2018, a surveillance study in Botswana evaluating adverse birth outcomes among women on different antiretroviral therapy (ART) regimens reported a 9-fold increase in the prevalence of NTDs among women who began treatment with dolutegravir (DTG) prior to conception (Zash et al., 2018). DTG is a second-generation Integrase Strand Transfer Inhibitor (INSTI) used to treat patients living with Human Immunodeficiency Virus (HIV) (Shah et al., 2014). In the Botswana Tsepamo Study, NTDs reported for 4/426 (.94%) infants exposed to DTG from conception included encephalocele, myelomeningocele, iniencephaly, and anencephaly. These findings raised significant concern among scientific and medical communities, prompting the World Health Organization (WHO) to review the existing data and reevaluate guidelines for use of DTG in pregnant women. When an unexpected NTD ‘safety signal’ is raised for a new medication, the potential role of maternal folate deficiency and other known risk factors are considered. However, due to the multifactorial nature of NTDs, additional elements that might tip the balance to create the ‘perfect storm’ can often be elusive.

In response to the safety alert, the Botswana Harvard AIDS Partnership conducted a larger follow-up study, and found that the prevalence of DTG-NTDs (5/1683 deliveries, .30%) was lower than in the original report, although still elevated compared to other ART regimens (.10%) (Zash et al., 2019). Notably, in all 5 of the NTD-affected pregnancies, DTG therapy was initiated more than 3 months prior to conception (Zash et al., 2022). The use of folic acid supplements did not differ across groups, and no other confounders were present. Based on this new evidence, and a risk-benefit assessment, the WHO again recommended DTG as the preferred first (and second)-line ART for all adults, including women of reproductive age and women who are pregnant or breastfeeding (WHO, 2019).

The preclinical embryo-fetal development (EFD) studies found no significant evidence of DTG-related maternal or developmental toxicity when pregnant animals were administered oral doses of DTG up to 1000 mg/kg/d (Stanislaus et al., 2020). Animals were exposed to DTG beginning on gestational day (GD) 6 and continuing until GD 17 (rats) or GD 18 (rabbits). DTG exposure in both species occurred post-conception, but prior to neurulation and organogenesis. The use of animal models for pre-market testing of new drugs is often a reliable predictor of potential adverse birth outcomes in humans (Koren et al., 1998). Experimental variables (genetic background, diet, husbandry, timing of exposure) in such studies are well-controlled. However, numerous confounding variables exist in human populations, making it difficult to evaluate and/or predict in a laboratory setting the complex interactions that may ultimately culminate in failure of neural tube closure. Post-marketing pharmacovigilance is therefore necessary to further evaluate the safety of new medications in pregnant women, and additional investigation is warranted when a safety signal is identified.

After the report from the Tsepamo Study was released, several laboratories conducted investigations using animal or cell-based models to examine potential adverse effects of DTG, most of which focused on the folate pathway. Studies in Madin-Darby canine kidney II cells did not find a significant DTG or INSTI class effect on inhibition of folate transport (Zamek-Gliszczynski et al., 2019). In contrast, DTG partially inhibited FOLR1-mediated uptake of folic acid in human placental trophoblast cells (Cabrera et al., 2019), and reduced FOLR1-mediated uptake in human choriocarcinoma cells (Gilmore et al., 2022). Significant mortality occurred in zebrafish embryos when DTG exposure was initiated at 3 h post-fertilization, but not when exposure began later in gastrulation-stage embryos, and mortality could be rescued by supplemental folic acid (Cabrera et al., 2019).

In 2 different human embryonic stem cell (hESC) lines, DTG exposure resulted in cytotoxicity and a dose-dependent loss of pluripotency (Smith et al., 2022). In a 3-dimensional (3D) hESC morphogenesis model, exposure to DTG caused altered growth and expression of genes involved in body patterning (Kirkwood-Johnson et al., 2021). DTG was also tested in a pluripotent P19C5 mouse embryonal carcinoma cell 3D morphogenesis model validated for the study of developmental toxicants (Marikawa et al., 2020; Warkus and Marikawa, 2017). When DTG exposure occurred within the first 1–2 days (corresponding to pre/early gastrulation), changes were observed in the expression of genes involved in axial patterning. A concentration-dependent inhibition of growth and axial elongation was also observed, but not when DTG exposure was initiated later in development (2–4 days) (Kirkwood-Johnson et al., 2021). In this study, supplementation with folic or folinic acid did not demonstrate a protective effect nor restore normal morphogenesis.

Additional studies were conducted using rodent models. Whole rat embryos were cultured *ex vivo* and exposed to DTG during the window of neurulation (GD 9–11] (Posobiec et al., 2021). DTG concentrations used were approximately 2 times higher than the plasma concentration reported for the maximum recommended human dose (MRHD) of 50 mg DTG twice daily, but no developmental toxicity or NTDs were observed (Posobiec et al., 2021). However, a limited number of studies in mice have been able to demonstrate a positive connection between DTG and NTDs. In C57BL/6J mice, 2.5 mg/kg/d oral DTG (co-formulated with tenofovir [TDF] and emtricitabine [FTC]) was found to yield peak plasma DTG concentrations (Cmax) that approximate human plasma concentrations. Mice were maintained on a folate-sufficient diet, and DTG exposure began at the time of conception (GD 0.5). This treatment regime yielded 5/150 NTD-affected litters (3.3%), with n = 1 NTD/affected litter (Mohan et al., 2021); however, no NTDs were observed in n = 111 litters from dams treated with a 5x higher dose. Another study in C3H/HeJ mice found 1 NTD affected pup in 17 litters examined (5.8%) when pregnant dams were given 50 mg/kg/d DTG oral gavage beginning on GD 0.5 (Bade et al., 2021). In pregnant C57BL/6J mice maintained on a folate-deficient diet administered oral 12.5 mg/kg/d DTG beginning on GD 0.5, modest changes in mRNA transcript levels of folate transporters (upregulation of *Rfc1* and downregulation of *Folr1*) were detected in GD 10.5 placentas (just after neural tube closure); however, when the mice were maintained on a folate-sufficient diet, a modest increase in *Folr1* mRNA occurred only at the 2.5 mg/kg/d DTG dose, and no changes were observed at the 5x higher dose (Gilmore et al., 2022).

Collectively, key findings from human, cell-based, and animal studies demonstrate an important connection between very early (pre-gastrulation) exposure to DTG and potential for disruption of morphogenesis, axial patterning, gastrulation, and neural tube closure. Nonetheless, studies evaluating a possible connection between DTG and folic acid rescue or altered transport of folic acid in different models have yielded variable results, suggesting the relationship between DTG and folate may be indirect, rather than direct. In published *in vivo* mouse models of DTG-NTDs, the DTG dose(s) used were calculated to approximate or slightly exceed human exposure based on established plasma levels in patients. However, the % of NTD-affected litters and the mean litter rate was small, which would make it difficult to detect with any certainty whether maternal folate supplementation *in vivo* has a protective effect.

In the current study we decided to focus on the potential role of a different micronutrient as a risk factor for DTG-NTDs, based on the known mechanism of action for the INSTI class of ART. DTG and other INSTIs chelate two magnesium (Mg^2+^) ions in the active site of the HIV integrase, inhibiting its catalytic activity and preventing incorporation of viral DNA into the host genome (Kawasuji et al., 2006; Barreca et al., 2009). Mg^2+^ is found in foods such as nuts, whole grains, and seeds, and is an important co-factor for hundreds of enzymatic reactions in the body (Mathew and Panonnummal, 2021; Jahnen-Dechent and Ketteler, 2012). In plasma, ∼60–70% of Mg^2+^ is ionized (Rosanoff et al., 2022), and homeostasis is regulated primarily by Mg^2+^ absorption in the small intestine and reabsorption in the kidneys (Schuchardt and Hahn, 2017; Claverie-Martin et al., 2021). Hypomagnesemia (<.7 mml/L) can lead to cardiac arrhythmias, and seizures (de Baaij et al., 2015) and is implicated in the pathogenesis of obesity, insulin resistance, and type 2 diabetes mellitus (T2DM) [reviewed in Pelczynska et al., 2022].

In humans, chronic latent hypomagnesemia is not uncommon, and undetected (subclinical) Mg^2+^ deficiency is prevalent in women of childbearing age worldwide (Dalton et al., 2016). Inadequate Mg^2+^ intake over a period of several months leads to slow depletion of serum Mg^2+^ that may not be recognized clinically, even in cases of severe hypomagnesemia (Elin, 1988). Analysis of NHANES (2003–2014) data revealed that women living with HIV in the United States not only had lower serum folate levels, but also lower intake of Mg^2+^ than women who were not infected (Thuppal et al., 2017). In the U.S., only ∼66% of prenatal vitamin supplements contain Mg^2+^, and only 5% of those on the market meet or exceed the 350–400 mg/day recommended daily amount (RDA) for pregnant women (Adams et al., 2021). Women of reproductive age living with HIV in resource-limited countries are likely to be at increased risk for undernutrition and consumption of poor-quality diets deficient in folate and other micronutrients, including magnesium (Darnton-Hill and Mkaru, 2015).

Two of the major transporters involved in regulating Mg^2+^ homeostasis in the body are the transient receptor potential melastatin (TRPM) divalent-selective ion channels TRPM6 and TRPM7 (Voets et al., 2004; Chubanov et al., 2005; Groenestege et al., 2006; Schlingmann and Gudermann, 2005; Schlingmann et al., 2007). In the mouse embryo, these 2 Mg^2+^ transporters also play critical non-redundant roles in cell movement during gastrulation and neurulation (Liu et al., 2011; Komiya et al., 2014; Komiya and Runnels, 2015; Komiya et al., 2017; Runnels and Komiya, 2020): *Trpm7* knockout mice initiate gastrulation but die *in utero* between GD 6.5–7.5 (Jin et al., 2008), while *Trpm6* knockout mice exhibit NTDs (Walder et al., 2009). Transport of adequate Mg^2+^ from maternal blood across the placenta to the developing embryo is essential for normal embryonic development and neural tube closure (Chubanov et al., 2016). In mice, Mg^2+^ deficiency or insufficiency during pregnancy causes placental abnormalities, growth restriction, and increased fetal mortality (Schlegel et al., 2015). In human studies, low maternal dietary intake of Mg^2+^ increased risk for spina bifida (Groenen et al., 2004) while, in contrast, periconceptional dietary intake of foods rich in magnesium decreased risk for NTDs (Shaw et al., 1999).

Based on the known mechanism of action for DTG (Mg^2+^ chelation) and the importance of Mg^2+^ in neural tube closure, we hypothesized that if DTG administration was initiated at conception, slow depletion of ionized Mg^2+^ in maternal plasma might occur over time, reducing the amount available for transport across the placenta as the pregnancy progressed. In dams unable to compensate and maintain Mg^2+^ homeostasis through increased dietary intake and/or increased renal reabsorption, the level of available Mg^2+^ might be inadequate to support normal neural tube closure. In addition, the unbound fraction of DTG that crosses the placenta might chelate Mg^2+^ present in coelomic or amniotic fluid and/or embryonic tissues. We hypothesized that in mice with pre-existing (subclinical) hypomagnesemia due to genetic variation and/or dietary Mg^2+^ insufficiency at the time of conception/initiation of DTG treatment, Mg^2+^ levels would be more likely to drop below a critical threshold before the onset of neurulation. In other words, in mice with underlying nutritional and/or genetic risk factors that compromise Mg^2+^ homeostasis but still allow embryogenesis to proceed normally, the addition of DTG might ‘tip the balance’ to create the perfect storm for development of NTDs.

To test our hypothesis concerning pre-existing perturbations in Mg^2+^ homeostasis as an underlying risk factor for DTG-NTDs, we selected 3 mouse strains on different genetic backgrounds that had inherently different basal plasma Mg^2+^ levels, ranging from low (subclinical hypomagnesemia) to high. In the first set of experiments, these 3 mouse strains were maintained on the same diet and administered the same dose of DTG. Litter outcomes were compared across strains for susceptibility to DTG-NTDs based on genetic variation in plasma and urine Mg^2+^ levels. In the second set of experiments, we placed mice on diets with different concentrations of Mg^2+^ (deficient, control, high) to examine the effect of reduced dietary Mg^2+^ intake on susceptibility to DTG-NTDs. In both experiments, plasma and urine Mg^2+^ levels were determined before mice were placed into timed mating. Dams were treated with vehicle or DTG by daily oral gavage, beginning on GD 0.5, and embryos examined for NTDs on GD 9.5. Plasma DTG levels were measured for pharmacokinetic (PK) analysis. In a third experiment, we analyzed whole-exome sequencing (WES) data from our inbred mouse strains to determine if the strain most susceptible to DTG-NTDs had variants in genes involved in Mg^2+^ homeostasis.

## 2 Materials and methods

### 2.1 Selection of mouse strains

Mouse strains were selected for testing based on predicted basal plasma levels of magnesium reported in the Jackson Laboratory Mouse Phenome Database (Yuan et al., 2008). The C3H/HeJ strain was selected for low (0.90 mml/L) serum Mg^2+^ and the C57BL/6J strain for high (1.19 mmol/L) serum Mg^2+^. These 2 strains were purchased from Jackson Labs (Bar Harbor, ME). The SWV and LM/Bc (on C3H background (Harris et al., 1984)) strains were also tested. These 2 inbred strains have been maintained as closed colonies by the van Waes lab for >20 years and used extensively for teratology studies.

### 2.2 Animal husbandry and timed matings

Breeding colonies and experimental mice were housed in Thoren micro-isolator cages with autoclaved corncob bedding and maintained on a 12-h light-dark cycle in climate-controlled AAALAC-accredited animal facilities at Creighton University. Mice were given *ad libitum* access to autoclaved water and pelleted irradiated rodent chow. For timed matings, nulliparous female mice were placed with a male overnight (1 male:2-3 females). The presence of a vaginal plug the next morning was considered GD 0.5. Plugged females were weighed, placed into a separate cage, and randomly assigned to a treatment group. All animal procedures were performed in accordance with the Public Health Service (PHS) policy on Humane Care and Use of Laboratory Animals and approved by the Creighton University Institutional Animal Care and Use Committee (IACUC).

### 2.3 Dolutegravir: Route of administration, dose selection, and gestational window of exposure

#### 2.3.1 Route and timing of administration

Dolutegravir (CAS 1051375-16-6, GSK1349572) was purchased from MedKoo Biosciences (Morrisville, NC). Pregnant mice were dosed with vehicle (dimethylsulfoxide: Solutol^®^: 50 mM *N*-methylglucamine in 3% mannitol [1:1:8, v:w:v]) (Moss et al., 2015) or DTG (500 mg/kg or 750 mg/kg) by daily oral gavage beginning on GD 0.5 and continuing until the day of sacrifice (GD 9.5). All treatments were administered in a volume of 0.1 mL/10 g bodyweight (bwt).

#### 2.3.2 Rationale for dose selection

According to the European Medicines Agency ICH S5 (R3). (2020) Guidelines on Reproductive Toxicology: Detection of Toxicity to Reproduction for Human Pharmaceuticals: “For small molecules, a systemic exposure representing a large multiple of the human AUC or Cmax at the maximum recommended human dose (MRHD) can be an appropriate endpoint for high dose selection. Doses providing an exposure in pregnant animals >25-fold the exposure at the MRHD are generally considered appropriate as the maximum dose for Developmental and Reproductive Toxicology studies and should be sufficient to detect the teratogenic hazard. Biological plausibility is assessed by comparison of pharmacologic mechanism of action with the known role of the target in development. This relationship is further strengthened by evidence that the finding is dose-related, whether characterized as increasing incidence or severity. In the absence of confounding maternal toxicity, the selection of a high dose for EFD studies that represents a >25-fold exposure ratio to human plasma exposure of total parent compound at the intended maximal therapeutic dose is therefore considered pragmatic and reasonably sufficient for detecting outcomes relevant for human risk assessment.” [ICH S5 (R3), 2020; Andrews et al., 2019] Based on the NOAEL (1000 mg/kg/d) and DTG dose range used in preclinical studies involving rabbits and rats (Stanislaus et al., 2020), and recommended appropriate high dose selection of >25-fold the MRHD for rodent EFD studies, we started with an oral test dose of DTG 750 mg/kg/d in the selected mouse strains. Blood samples were collected from pregnant and non-pregnant mice for pharmacokinetic (PK) determination of Cmax and AUC to confirm the DTG exposure ratio relative to human plasma.

### 2.4 Experimental diets

The SWV and LM/Bc breeding colonies have been maintained on Envigo Rodent Chow #8904 (a soy protein-based irradiated diet) for >20 years. C3H/HeJ and C57BL/6J mice (6–10 weeks old) were immediately placed on Envigo #8904 upon arrival at Creighton University and were maintained on this diet for a minimum of 1 month prior to analysis of basal plasma and urine cations. The National Research Council (NRC) estimate for the minimum Mg^2+^ requirement in adult mice is 0.5 ppm of diet (0.05%). Magnesium in standard rodent diets ranges between 2–3 g mg/kg; the Envigo #8904 soy diet contains .3% Mg^2+^ by weight (5 times the NRC requirement) and was therefore considered a ‘high’ Mg^2+^ diet. Sources of Mg^2+^ in the soy diet include magnesium oxide (∼20%) and Mg^2+^ found naturally in grain ingredients (∼80%); it was therefore not possible to remove Mg^2+^ to create a soy-based version that was Mg^2+^ deficient. To test the hypothesis that low maternal plasma Mg^2+^ prior to conception would increase susceptibility to DTG-induced NTDs, it was necessary to switch to casein-based purified ingredient diets with defined levels of Mg^2+^ added back. The following casein-based purified ingredient diets were selected as experimental diets: TD.120,574 high Mg^2+^ diet (.3% Mg^2+^ oxide added to match the .3% high Mg^2+^ soy-based diet); TD.98341 control Mg^2+^ diet with 0.05% Mg oxide added back to match the NRC requirement; and TD.93106 Mg^2+^-deficient diet (<.003%, no Mg^2+^ added back). The purified casein diets met or exceeded the NRC requirements for all other minerals and vitamins, including folic acid (Supplementary Table S1: Diet Comparison). Thus, the 4 experimental diets were as follows: 1) high Mg^2+^ (.3%) soy diet; 2) high Mg^2+^ (3%) casein diet; 3) control Mg^2+^ (.05%) casein diet; and 4) deficient Mg^2+^ (<.003%) casein diet. C57BL/6J mice placed on severely Mg^2+^-deficient diets (<.003% Mg^2+^) demonstrate a significant decrease in plasma Mg^2+^ within 10–14 days, although they continue to appear healthy (Bardgett et al., 2005). Our initial DTG teratology studies were performed in mice on the high Mg^2+^ soy diet. However, our objective in the second set of experiments was to reduce pre-conception plasma Mg^2+^ levels prior to DTG exposure. To accomplish this objective, weaned female mice were placed on either the high Mg^2+^ casein diet or one of the 2 casein-based experimental diets with reduced Mg^2+^ (control or deficient) for a minimum of 2 weeks. After this minimum period of acclimation to the experimental casein-based diets, blood and urine samples were collected for Inductively Coupled Plasma Mass Spectrometry (ICP-MS) determination of Mg^2+^ (and other cations).

### 2.5 Urine collection (metabolic cages)

Non-pregnant female mice of each strain were placed into metabolic cages in groups of 3 mice per cage (n = 3 cages of each strain/diet) and allowed to acclimate for 24 h before urine collection began. Following adaptation, 24-h pooled urine samples were collected from each cage for 3 consecutive days to determine strain and/or diet-induced differences in urine cations. The volume of urine collected for each 24-h period was recorded, and samples were frozen at −80 °C for ICP-MS analysis.

### 2.6 Blood collection

Baseline blood samples were collected via cheek bleed (submandibular vein) from all animals prior to timed matings, and again on GD 9.5. A Goldenrod sterile lancet (MEDIpoint, Inc., Mineola, NY) was used to lance the submandibular vein, and 3-4 drops (∼100 µL) of blood was collected into a Microvette CB 300 Lithium Heparin plasma separator tube (SARSTEDT AG and Co., Germany) and then centrifuged at 2000 *g* for 8 min. Plasma samples were frozen at −80 °C for LCMS analysis of DTG or ICP-MS determination of cations.

### 2.7 Embryo harvest and phenotyping

Pregnant dams were anesthetized with isoflurane and sacrificed by cervical dislocation on GD 9.5. The uterus was removed and examined under a dissecting microscope; individual implants were separated, assigned a number, and placed in ice-cold phosphate-buffered saline. Embryos were examined and the phenotype reported as normal (GD 9.5, fully turned, Theiler stage 14, cranial neural tube closed), resorption, developmental delay (not fully turned, Theiler Stage 12–13, open neural folds), or NTD (GD 9.5, fully turned, exencephalic). Whole embryos were photographed under a Nikon SMZ 1500 stereomicroscope mounted with an AmScope™ 18MP USB3.0 digital camera. Placentas (decidua basalis) and embryos were fixed in 10% formalin or frozen at −80°C.

### 2.8 Liquid chromatography—Mass spectrometry (LC-MS/MS): Determination of plasma DTG concentration

DTG detection and quantification was performed at Creighton University using a previously published method (Bennetto-Hood et al., 2014) with minor modifications. Briefly, a seven-point calibration curve ranging from 0.1 to 100 μg/mL was challenged with quality control vials at known concentrations of 5, 20, and 80 μg/mL. All controls and calibrators with known drug concentrations were added to DTG-free mouse plasma. To initiate protein precipitation, 120 µL of acetonitrile containing 10 ng/mL of d5- DTG internal standard (Cayman Chemical, Ann Arbor, MI) was added to 20 µL mouse plasma (calibrators, controls, unknown samples), and mixed for 2 min on an orbital shaker (1500 rpm), followed by centrifugation at 2,655 *g* for 5 min. Finally, 20 µL of supernatant was transferred into a vial containing 120 µL of 1 mg/mL EDTA (pH 8) with 0.1% formic acid. LC-MSMS separation was achieved using Agilent 1200 liquid chromatography devices and an AB Sciex 3200 Q-Trap mass spectrometer. The mobile phase consisted of an isocratic gradient of 0.1% formic acid in water:0.1% formic acid in acetonitrile (60:40) with a flow rate of 0.475 mL/min across an XBridge C18 column (Waters, Milford, MA, United States). The mass spectrometer identified DTG (m/z = 420.2 (277.2) and d5-dolutegravir (m/z 425.2 (277.2) in MRM scan mode with ESI positive polarity. All solvents used were HPLC grade and above. Linear best fit curves of calibrators allowed for quantification of control and unknown sample concentrations.

#### 2.8.1 Pharmacokinetics

Blood was collected from separate groups of n = 3 non-pregnant LM/Bc female mice/time point for each of 10 different time points (0.5, 1, 2, 3, 4, 6, 8, 16, 20, and 24 h) following oral gavage administration of a single dose of DTG (750 mg/kg). Plasma DTG levels were determined by LC-MS/MS and PK Solver v2.0 (Zhang et al., 2010) was used to determine Cmax, Tmax, t½, and AUC. To evaluate potential differences in DTG pharmacokinetics (PK) between non-pregnant females (single oral dose DTG750) and pregnant females (10 doses DTG750, daily oral gavage from E0.5–E9.5), pregnant mice were given the last DTG dose on E9.5 and blood samples were collected at 12 different time points over a period of 24 h after that dose.

### 2.9 ICP-MS determination of plasma and urine cations

ICP-MS analyses of plasma and urine Mg^2+^ (and 17 other cations) were performed by Dr. Javier Seravalli at the Spectroscopy and Biophysics Core Facility, Redox Biology Center, University of Nebraska-Lincoln. These analyses used an Agilent Technologies ICP-MS 7500cs and an ESI SC-4 high-throughput autosampler, and the method was optimized for the analysis of eighteen elements (including ^24^Mg and ^44^Ca). Each biological sample was run in triplicate.

### 2.10 Statistical analysis

Strains and treatments were compared by 2-way ANOVA followed by *post hoc* analyses. Pairwise comparisons of the proportion of affected embryos in each litter between strains and treatments were evaluated by the Fisher least significant difference method. Index value pairwise comparisons were evaluated using the Kruskal–Wallis nonparametric test in Sigma XL (v9.0). Comparisons of cation levels in urine and plasma between strains and between diets as well as the effect of DTG on the incidence of NTDs in LM/Bc dams was performed by one-way ANOVA followed by Dunnett’s multiple comparisons test using GraphPad Prism version 9.5.0 for Windows. The level of significance *p* < 0.05 was used for all analyses. DTG pharmacokinetics were evaluated by noncompartmental analysis (NCA) using PKsolver (Zhang et al., 2010).

### 2.11 Whole-exome sequencing, alignment, detection of genetic variants

DNA from tail snips of LM/Bc and SWV mice were extracted using the Wizard Genomic DNA Purification Kit (Promega Corporation, Madison, WI). Exonic regions were enriched for one mouse of each strain using the SureSelect XT Mouse All Exon kit (Agilent Technologies, Santa Clara, CA) according to the manufacturer’s recommendations. This kit targets 221,784 exons within 24,306 genes, with a total capture size of 49.6 MB. Prepared libraries were sequenced 2x100nt on an Illumina HiSeq at the David H. Murdock Research Institute (Duke University) in 2012. The resulting data was aligned against the NCBI37 mouse reference genome, the alignments cleaned, and the variants called, except without use of the contamination threshold parameter or allele phasing. To reduce artifacts and false positives, variants were required to have a read depth of at least 4, fewer than 10% of reads with mapping quality of 0, and a quality-by-depth of at least 7.

#### 2.11.1 Identification of genetic variants involved in Mg^2+^ homeostasis

Variants were lifted over to the GRCm39 (C57BL/6J) reference genome using the Ensembl Assembly Converter tool and annotated using the Variant Effect Predictor (VEP) tool. To identify potential variants that might explain differences in basal plasma and urine Mg^2+^ and susceptibility to DTG–NTDs between the different mouse strains, moderate (missense) and high (nonsense, splicing, frameshifting, and start-loss) risk variants in known magnesium pathway homeostasis genes were examined. The set of magnesium homeostasis genes was assembled using a union of OMIM and Clinvar genes. A list of the 35 candidate genes analyzed is given in Table 1.

**TABLE 1:** Candidate genes involved in magnesium homeostasis.

*Apc*	*Clcnkb*	*Egf*	*Hip14l*	*Mmgt2*	*Nipal1*	*Slc41a1*
*Atp13a2*	*Cldn16*	*Egfr*	*Hnf1b*	*Mrs2*	*Nipal4*	*Slc41a2*
*Atp13a4*	*Cldn19*	*Fam111a*	*Kcna1*	*Mt-t1*	*Pcbd1*	*Trpm6*
*Bsnd*	*Cnmm2*	*Fyxd2*	*Kcnj10*	*Nipa1*	*Sars2*	*Trmp7*
*Casr*	*Cnnm2*	*Hip14*	*Mmgt1*	*Nipa2*	*Slc12a3*	*Xk*

#### 2.11.2 Primer design, Sanger validation

Primers for Sanger validations were designed using Primer3web (v 4.1.0). DNA was extracted from tail snips using the PureLink™ Genomic DNA Mini Kit (Invitrogen). Samples were amplified using the Phusion Flash High-Fidelity PCR Master Mix (Thermo Fisher) and 1 µM of primers. PCR was denatured at 98 °C for 10 s, followed by 35 cycles of 98 °C for 1 s, 48 °C for 5 s, and 72 °C for 15 s, and a final extension of 72 °C for 1 min. Products were cleaned using AMPureXP beads (Beckman Coulter) at 1.8X, eluted in water, and quantified using a Qubit BR dsDNA kit (Invitrogen). Sanger sequencing was performed at Genewiz/Azenta Life Sciences and consensus calling was manually checked from chromatograph traces using GeneSnap Viewer v5.3.2.

## 3 Results

### 3.1 Strain comparison: Basal plasma and urine cations on the high Mg^2+^ soy natural ingredient diet

#### 3.1.1 Strain differences in basal plasma cations

**FIGURE 1:**
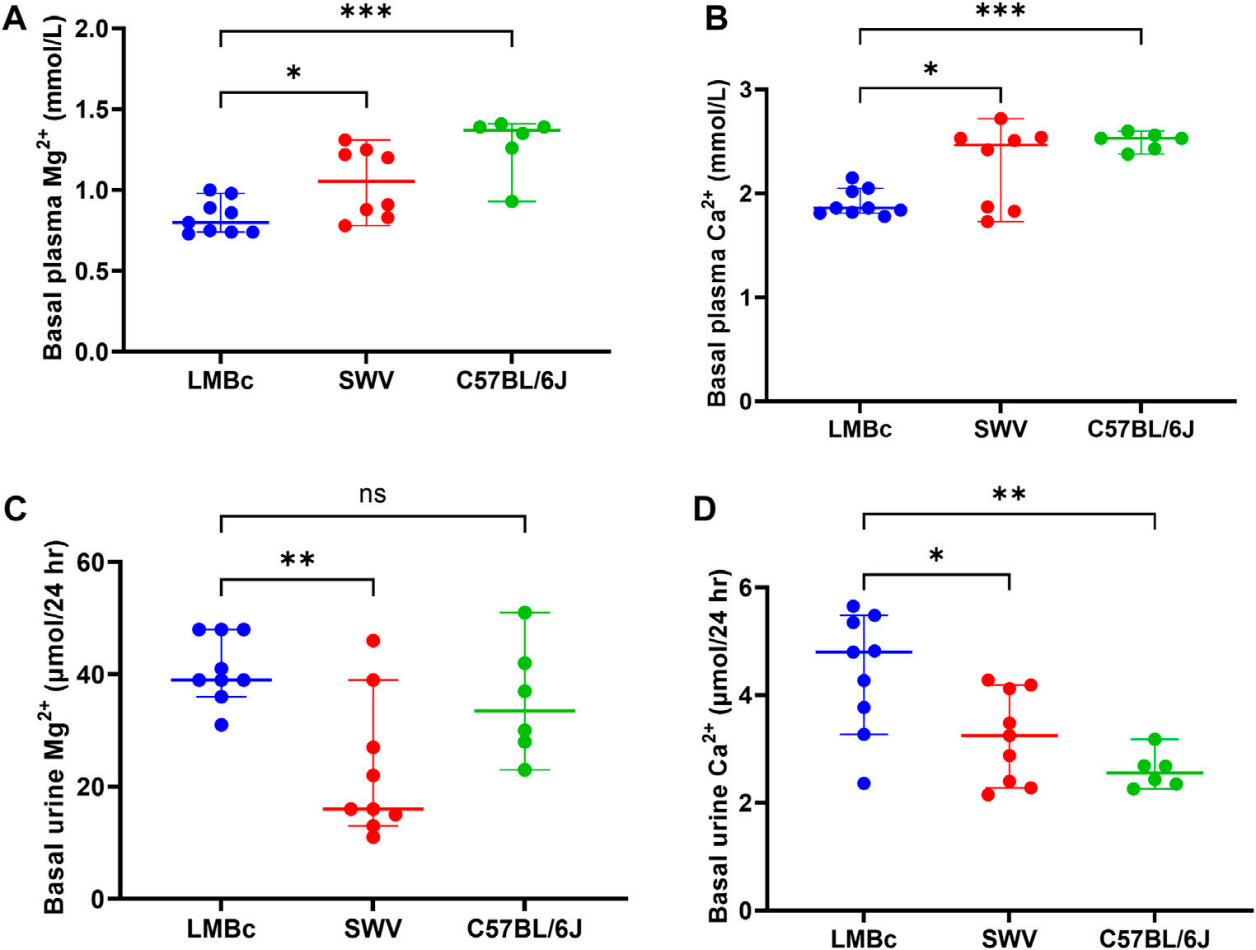
Strain comparison: Basal plasma and urine cations. The results of the strain comparison for **(A)** basal plasma Mg^2+^ and **(B)** basal plasma Ca^2+^ are shown (median mmol/L ± 95% CI). LM/Bc mice have inherently lower plasma Mg^2+^ and Ca^2+^ than either the SWV (*p < 0.05) or the C57BL/6J strain (***p < 0.001). The results of the strain comparison for **(C)** basal urinary excretion of Mg^2+^ and **(D)** basal urinary excretion of Ca^2+^ **(D)** are also shown (µmol/24 h ± 95% CI). Urinary excretion of Mg^2+^ in LM/Bc was significantly higher than SWV (**p < 0.01) but did not differ from C57BL/6J. However, urinary Ca^2+^ excretion in LM/Bc mice was significantly higher than in both SWV (*p < 0.05) and C57BL/6J (**p < 0.01) mice.

After a minimum of 4 weeks on the high Mg^2+^ soy diet, average basal plasma Mg^2+^ levels were determined for each of the 4 mouse strains (n = 9 non-pregnant females/strain). The results are shown in Figure 1A. As predicted, basal plasma Mg^2+^ values (average ± SEM) were lowest for C3H/HeJ (0.78 ± 0.03, not shown) and LM/Bc (0.83 ± 0.03), and not significantly different from one another. Moreover, basal plasma Mg^2+^ for LM/Bc was significantly lower than SWV (1.05 ± 0.08) and C57BL6/J (1.29 ± 0.07). Based on these results, C3H/HeJ and LM/Bc were considered to have ‘low’, SWV ‘intermediate’, and C57BL6/J ‘high’ basal plasma Mg^2+^ levels.

Similar results were obtained when comparing basal plasma Ca^2+^ across strains (Figure 1B). Plasma Ca^2+^ (average ± SEM) was not significantly different between C3H/HeJ (1.91 ± 0.09) and LM/Bc (1.91 ± 0.04). However, plasma Ca^2+^ was significantly lower in LM/Bc when compared to SWV (2.27 ± 0.14) with ‘intermediate’ and C57BL6/J (2.51 ± 0.03) with ‘high’ plasma Ca^2+^ levels.

#### 3.1.2 Strain differences in basal urinary cations

After a minimum of 4 weeks on the high Mg^2+^ soy diet, basal urinary excretion of Mg^2+^ (Figure 1C) and Ca^2+^ (Figure 1D) were determined for the 4 mouse strains. Although both the C3H/HeJ and LM/Bc strains had low plasma Mg^2+^ on this diet, they differed in urinary Mg^2+^ excretion. The 24 h urinary Mg^2+^ output (µmol/24 h ± SEM) in LM/Bc mice (41.07 ± 2.03) was significantly higher compared to that in C3H/HeJ (28.08 ± 3.61, not shown) and SWV mice (22.82 ± 4.12). Meanwhile, in C57BL/6J mice, urinary Mg^2+^ was high (35.06 ± 4.21), and not significantly different from LM/Bc. However, unlike LM/Bc mice, C57BL/6J mice were able to maintain high basal plasma Mg^2+^ levels despite elevated urinary Mg^2+^ excretion (Figure 1A).

Urinary Ca^2+^ excretion (µmol/24 h ± SEM) was also significantly higher in LM/Bc (4.42 ± 0.37) compared to SWV (3.23 ± 0.28) and C57BL/6J (1.73 ± 0.44) (Figure 1D) but did not differ from the C3H/HeJ strain (3.92 ± 0.25, not shown). LM/Bc and C3H/HeJ mice both have low basal plasma Mg^2+^ and Ca^2+^ levels; the major difference is that only the LM/Bc strain exhibits hypermagnesuria (Figure 1C). Continued high urinary Mg^2+^ output in the LM/Bc strain despite low basal plasma Mg^2+^ levels on the high Mg^2+^ soy diet suggests renal Mg^2+^ wasting and an imbalance in Mg^2+^ homeostasis.

### 3.2 Strain comparison: DTG-NTD susceptibility on high Mg^2+^ soy diet

Based on basal plasma Mg^2+^ levels, the LM/Bc strain was predicted to be susceptible to DTG-NTDs, whereas the SWV and C57BL/6J strains were predicted to be more resistant. The strain comparison with respect to NTD susceptibility on the high Mg^2+^ soy diet and DTG750 treatment is shown in Table 2. The dam is considered the experimental unit; comparisons are between litter means. The mean litter rate was calculated by averaging the % of embryos in each litter that had an NTD. Any litter with one or more exencephalic embryo(s) was considered ‘affected’. No NTDs were observed in any vehicle-treated litter, regardless of strain. No NTDs were observed in C57BL/6J litters (n = 10) treated with DTG750. However, 3 pups with exencephaly were observed in 1/13 litters (7.6%) from DTG750-treated SWV dams, and a total of 28 pups with exencephaly (out of 166 total implants) were observed in 12/19 (63.2%) litters from DTG750-treated LM/Bc dams (1–6 NTDs/affected litter). The number of affected litters following exposure to DTG750 was significantly higher in the LM/Bc strain compared to either the SWV or C57BL6/J strain (*p* < .01). Unexpectedly, there were no significant differences between strains in the number of resorptions or developmental delays following exposure to DTG750.

**TABLE 2:** Strain comparison: NTDs ON HIGH Mg2+ SOY DIET x DTG750. Pregnant dams from three different mouse strains with inherently different basal plasma Mg2+ levels were maintained on a high Mg2+ (0.3%) soy diet and treated with oral vehicle or DTG (750 mg/kg/d) from the day of conception (GD 0.5) until GD 9.5. The strains were compared for susceptibility to DTG-NTDs. The dam is considered the experimental unit and comparisons are between litter means. The mean litter rate was calculated by averaging the % of embryos in each litter that had an NTD. A litter is considered affected if at least 1 embryo presented with exencephaly (NTD). Statistical analysis was done by 2-way ANOVA and *post hoc* analysis (Fisher least significant difference method) or by the Kruskal–Wallis test for nonparametric analyses of index values, both using the software Sigma XL (https://www.sigmaxl.com/). Within each variable, the highlighted numbers and asterisks indicate statistical differences in pairwise comparisons (*α* > 0.99).

	LMBc		SWV		C57BL/6J	
Mean (SD)	VEH	DTG750	VEH	DTG750	VEH	DTG750
Implants per litter	8.40 (1.65)	8.74 (2.47)	14.17 (1.03)^**^	13.15 (2.12)^**^	8.82 (1.47)	9.80 (1.48)
Resorptions per litter	0.90 (0.88)	0.95 (1.27)	1.25 (1.29)	0.62 (0.87)	0.82 (1.08)	0.50 (0.85)
Total viable embryos	7.50 (1.08)	7.79 (2.76)	12.92 (1.08)^**^	12.54 (2.37)^**^	8.00 (2.19)	9.30 (1.77)
Dev. delayed	0.00 (0.00)	0.58 (0.96)	0.25 (0.45)	0.31 (0.48)	0.09 (0.30)	0.20 (0.42)
NTDs	0.00 (0.00)	1.47 (1.74)^**^	0.00 (0.00)	0.23 (0.83)	0.00 (0.00)	0.00 (0.00)
Index Values Average % (SD)						
Resorptions/implants	9.6% (8.87)	11.5% (15.75)	8.5% (8.50)	5.0% (7.62)	10.8% (14.81)	5.3% (9.30)
Dev delay/implants	0.0% (0.00)	8.1% (17.76)	1.7% (3.16)	2.0% (3.19)	1.0% (3.35)	1.9% (4.15)
NTDs/implants	0.0% (0.00)	17.7% (20.63)^**^	0.0% (0.00)	1.6% (5.94)	0.0% (0.00)	0.0% (0.00)
NTDs/embryos	0.0% (0.00)	20.9% (23.65)^**^	0.0% (0.00)	1.8% (6.40)	0.0% (0.00)	0.0% (0.00)
Affected litters %	0.0%	63.2%^**^	0.0%	7.7%	0.0%	0.0%
Number of litters	10	19	12	13	11	10

**FIGURE 2:**
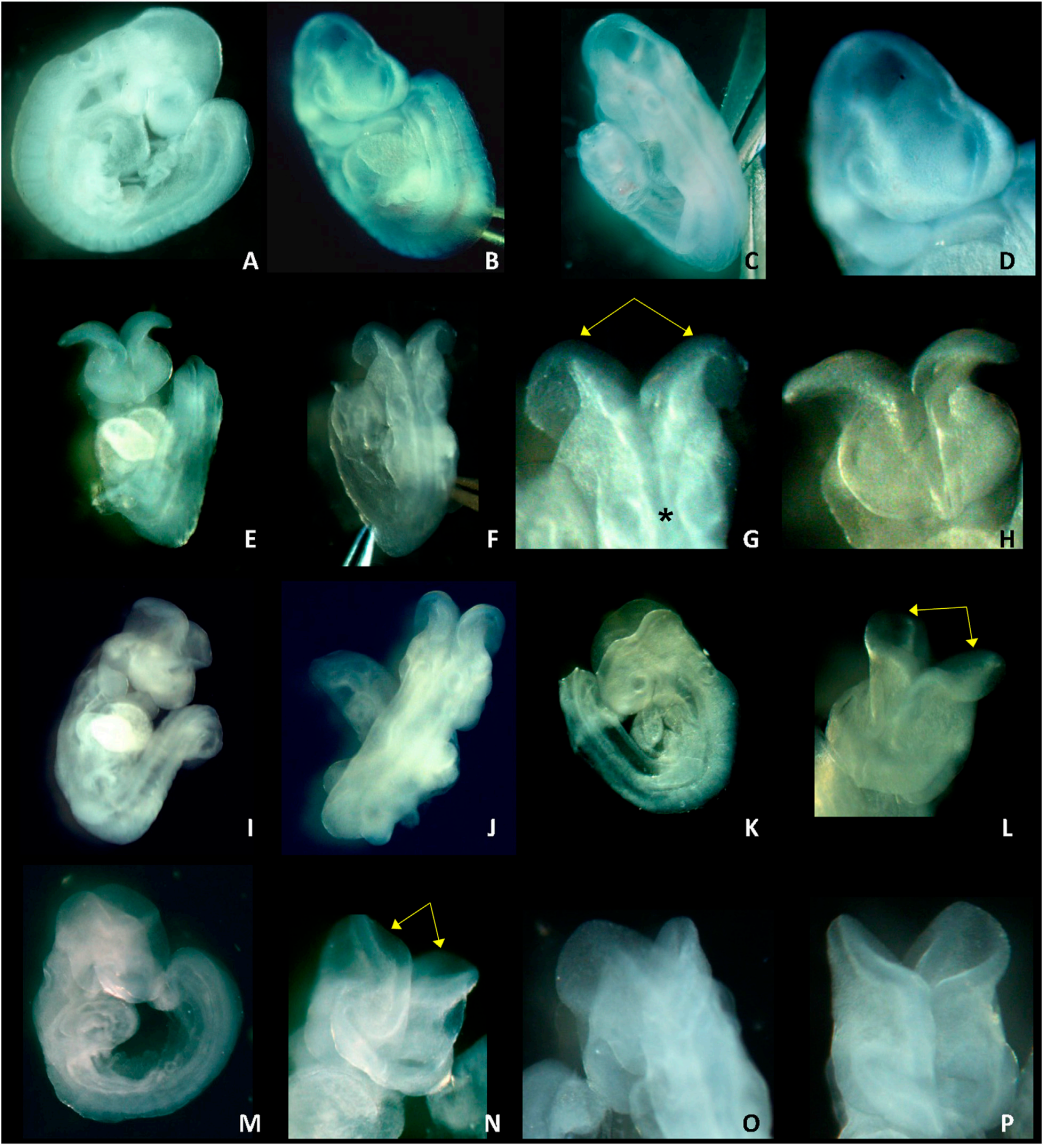
NTDs IN GD 9.5 LM/Bc EMBRYOS ON HIGH Mg^2+^ SOY DIET x DTG750. Photos of GD 9.5 embryos from LM/Bc dams on high Mg^2+^ soy diet treated with vehicle (first row) or from dams treated with DTG750 (second, third and fourth rows) are shown. First row: normal GD 9.5 embryos with closed neural tube from vehicle treated litter **(A)** side view of normal, fully turned GD 9.5 embryo; **(B)** front (rostral) view, normal; **(C)** back (caudal) view, normal; and **(D)** close-up of front view. The remaining three panels (rows) show representative exencephalic GD 9.5 embryos from litters treated with DTG750. Second row: Photos **(E—G)** are of the same GD 9.5 exencephalic embryo: **(E)** front view showing open (everted) cranial neural folds; **(F)** caudal view of the same embryo; **(G)** close-up of caudal view showing that from the rhombic lip (boundary between spinal neural tube and hindbrain, denoted by asterisk*) forward, the neural folds are open and diverge away from one another (yellow arrows); **(H)** close-up of rostral view. Third row: **(I, J)** rostral and caudal view of a GD 9.5 embryo with open cranial neural folds **(K)** side view and **(L)** close-up rostral view of another GD 9.5 embryo with open cranial neural folds (yellow arrows) that diverge and bend outward bilaterally at the dorsolateral hinge points. Bottom row: **(M–P)** different views of an exencephalic GD 9.5 embryo **(M)** side view; **(N)** front (rostral) view of open cranial neural folds (yellow arrows); **(O)** back (caudal) view; **(P)** close-up front view.

Photos of GD 9.5 LM/Bc embryos harvested from dams on the high Mg^2+^ soy diet treated with vehicle or DTG750 are shown in Figure 2. Vehicle-treated GD 9.5 embryos are fully turned, and the cranial neural tube is closed (Figures 2A–D). DTG750-treated GD 9.5 embryos are fully turned but have open cranial neural tubes (exencephaly) in which the neural folds bend outward (bilaterally) at the dorsolateral hinge points. Divergence of the neural folds begins at the rhombic lip (the boundary between the cervical spine and the hindbrain) and extends rostrally, including the boundary of the midbrain with the caudal forebrain, demonstrating failure of closure site 2 (Figure 2E–P).

No NTDs were observed in LM/Bc mice on the high Mg^2+^ soy diet when pregnant dams were treated with DTG 500 mg/kg/d (DTG500). Of the n = 7 litters examined at this lower dose, all 62 viable embryos were developmentally normal, fully turned GD 9.5 embryos with a closed cranial neural tube (71 implants, 9 resorptions, no developmental delays, 62 normal). Additional DTG doses on this diet will need to be tested to definitively determine the NOAEL.

### 3.3 DTG pharmacokinetics

The results of the LC-MS/MS pharmacokinetic analyses for plasma DTG in non-pregnant and pregnant LM/Bc mice on the high Mg^2+^ soy diet are shown in Table 3. The Cmax and AUC are compared to values reported from human studies in which non-pregnant participants received a single dose of DTG 50 mg (Min et al., 2010). The most prescribed dose for DTG is 50 mg/d, but the maximum human recommended dose (MHRD) is 50 mg twice a day (100 mg/d). Values for a single dose of DTG 100 mg (Min et al., 2010) and DTG 100 mg × 7d (Wang et al., 2019) are shown for comparison. PK values shown for pregnant women were from the second trimester (50 mg/d) (Mulligan et al., 2018). Using both the Cmax and AUC for comparison, a single dose of DTG750 in non-pregnant mice is less than the target goal of >25x the MHRD. In pregnant mice, multiple doses of DTG750 give a Cmax of 35.3x human values for the 50 mg/d dose in pregnant women, but an AUC of only 17.8x. Although Tmax is the same in pregnant mice and women (2 h) following oral dosing, DTG was metabolized more rapidly in mice; the t½ in mice is considerably shorter (4.4 h compared to 11 h in women) resulting in a reduced multiplication factor for AUC.

**TABLE 3:** Pharmacokinetics: Plasma DTG in LM/Bc MICE on HIGH Mg2+ SOY DIET. Published pharmacokinetic (PK) parameters (Cmax, AUC, t½, and Tmax) for single or multiple doses of DTG in non-pregnant and pregnant humans are compared to the PK parameters in LM/Bc mice receiving a single oral dose (non-pregnant) or multiple doses (pregnant) of DTG. The maximum human recommended dose (MHRD) for DTG is 50 mg twice a day (100 mg/d). Female LM/Bc mice were maintained on high Mg^2+^ soy diet. Non-pregnant females were administered a single oral dose of DTG (750 mg/kg/d) and pregnant females were administered the same oral dose (DTG750) daily (from GD 0.5 to GD9.5). For developmental toxicology studies, the goal is to provide a systemic exposure >25x the MHRD (Cmax or AUC) [ICH S5 (R3)]. In pregnant LM/Bc mice the Cmax approximates 35x the Cmax in pregnant women (second trimester) taking oral DTG (50 mg/d); however, in the same comparison, the AUC in pregnant mice approximates only 18x the AUC in pregnant women because the DTG t½ is much shorter in mice.

Mouse (DTG750 mg/kg, oral gavage)			Human	Mouse (multiplication factor)
PARAMETER	UNIT	VALUE		
**Cmax**				
Non-pregnant (single dose DTG750)	µg/mL	132.98	4.6 (50 mg single dose)^1^	28.9 x
			6.7 (100 mg × 7d)^2^	19.8 x
			8.1 (100 mg single dose)^1^	16.4 x
Pregnant (10 daily doses DTG750) GD 0.5—GD 9.5	µg/mL	127.89	3.62 (2.57–4.63)	**35.3 x**
			(2nd trimester, 50 mg/d)^3^	
**AUC**_**0-24**_				
Non-pregnant (single dose DTG750)	µg*h/mL	1299.36	70.8 (50 mg single dose)^1^	18.4 x
			92.3 (100 mg × 7d)^2^	14.1 x
			131 (100 mg single dose)^1^	9.9 x
Pregnant (10 daily doses DTG750) GD 0.5—GD 9.5	µg*h/mL	846.55	47.6 (33.4–63.7) (2nd trimester, 50 mg/d)^3^	**17.8 x**
**t½**				
Non-pregnant (single dose DTG750)	h	6.30 h	13–14 h (50 mg single dose)^1^	
Pregnant (10 daily doses DTG750)	h	4.40 h	11 h (2nd trimester, 50 mg/d)^3^	
**Tmax**				
Non-pregnant (single dose DTG750)	h	2.00 h	1.25 h (50 mg single dose)^1^	
Pregnant (10 daily doses DTG750)	h	2.00 h	2 h (2nd trimester, 50 mg/d)^3^	

^1^ (Min et al., 2010).

^2^ (Wang et al., 2019).

^3^ (Mulligan et al., 2018).

### 3.4 Plasma and urine Mg^2+^ on purified-ingredient casein diets with defined Mg^2+^ concentrations

To determine the role of reduced or deficient maternal dietary Mg^2+^ intake on DTG-NTD susceptibility, newly weaned female LM/Bc mice (from lactating dams maintained on the high Mg^2+^ soy diet) were placed on casein-based purified ingredient experimental diets with defined Mg^2+^ concentrations as previously described.

#### 3.4.1 Impact of reduced dietary Mg^2+^ intake on plasma and urine Mg^2+^ in LM/Bc mice

Non-pregnant LM/Bc mice were placed on the 3 experimental casein-based diets to evaluate the impact of reduced dietary Mg^2+^ intake on plasma and urine Mg^2+^ levels prior to timed matings.

The first group of female LM/Bc mice (n = 5) placed on the deficient Mg^2+^ casein diet all died unexpectedly within 5–7 days. They appeared active and healthy prior to being found dead in their cage. Necropsy did not reveal any remarkable findings. Low blood levels of Mg^2+^ are known to cause cardiac arrhythmias and seizures. Dietary Mg^2+^ insufficiency coupled with renal Mg^2+^ wasting in the LM/Bc mice likely resulted in a severe drop in plasma Mg^2+^ and death due to a sudden cardiac event. Due to this unforeseen outcome, it was not possible to move forward with experiments examining the combined effect of DTG and Mg^2+^-deficient diet in LM/Bc mice. Instead, LM/Bc females were placed only on the high or control Mg^2+^ casein diets.

Plasma and urine Mg^2+^ concentrations were measured in female LM/Bc mice (n = 9) on the high Mg^2+^ soy diet at the time of weaning and then again in the same group of mice after 2 weeks (15 days) on the high Mg^2+^ casein diet. Another group of LM/Bc mice (n = 6) was placed on the control Mg^2+^ casein diet at the time of weaning and had plasma and urine Mg^2+^ measured after 2 weeks (15 days) and again after 8 weeks (60 days). LM/Bc mice maintained on the control Mg^2+^ casein diet for 8 weeks continued to appear healthy. It was determined that additional LM/Bc mice could safely be placed on this diet for up to 8 weeks and used for timed matings.

##### 3.4.1.1 LM/Bc Plasma Mg^2+^

**FIGURE 3:**
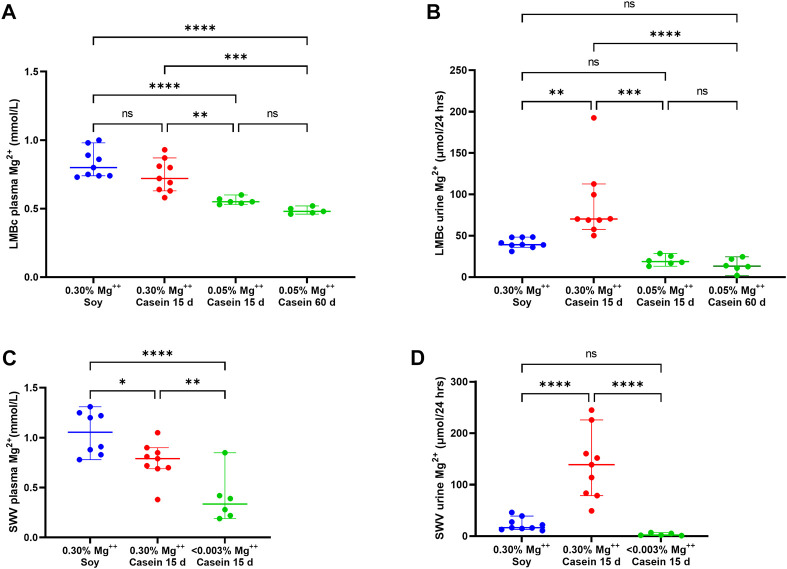
Impact of reduced dietary Mg^2+^ on plasma and urine Mg^2+^ in LM/Bc and SWV mice. The impact of reduced dietary intake of Mg^2+^ on plasma Mg^2+^ levels (median mmol/L ± 95% CI) in LM/Bc mice are shown in **(A)**. Plasma samples were evaluated in weaned females on high Mg^2+^ (0.3%) soy diet (blue dots) and 15 days after they were placed on the high Mg^2+^ (0.3%) casein diet (red dots). There was no significant change in plasma Mg^2+^ between the 2 high Mg^2+^ (soy and casein) diets. However, after LM/Bc mice were placed on the control Mg^2+^ (0.05%) casein diet for 15 days or 60 days (green dots), plasma Mg^2+^ dropped significantly when compared to either of the high Mg^2+^ diets. For all plots in Figure 3 (*p < .0.05, **p < 0.01, ***p < 0.01, ****p < 0.0001). The impact of reduced dietary intake on urinary excretion of Mg^2+^ (µmol/24 h ± 95% CI) for the corresponding diets and time points in LM/Bc mice is shown in **(B)**. After 15 days on the high Mg^2+^ casein diet (red dots), urinary Mg^2+^ excretion was significantly higher than on either the high Mg^2+^ soy diet (blue dots) or the control Mg^2+^ casein diet (green dots). After 15 days or 60 days on the control Mg^2+^ (0.05%) casein diet, urinary Mg^2+^ output was similar and not significantly different from the high Mg^2+^ soy diet. The impact of severely reduced dietary intake of Mg^2+^ on plasma Mg^2+^ levels (median mmol/L ± 95% CI) in SWV mice is shown in **(C)**. Plasma samples were evaluated in weaned females on high Mg^2+^ (0.3%) soy diet (blue dots) and again 15 days after they were placed on the high Mg^2+^ (0.3%) casein diet (red dots); somewhat unexpectedly, plasma Mg^2+^ levels decreased on the high Mg^2+^ (0.3%) casein diet (*p* < .05). After 15 days on Mg^2+^ deficient (<0.003%) diet, plasma Mg^2+^ levels dropped significantly (purple dots) relative to either of the high Mg^2+^ diets. The impact of reduced dietary intake on urinary excretion of Mg^2+^ (µmol/24 h ± 95% CI) for the corresponding diets and time points in SWV mice is shown in **(D)**. Like the trend observed in LM/Bc mice, urinary Mg^2+^ excretion in SWV mice increased significantly after 15 days on the high Mg^2+^.

The results for diet effect on plasma Mg^2+^ levels are shown in Figure 3A. In the LM/Bc strain, there was a slight but insignificant decrease in plasma Mg^2+^ (mmol/L ± SEM) when mice on the high Mg^2+^ soy diet (0.83 ± .04) were placed on the high Mg^2+^ casein diet for 15 days (0.74 ± .04). LM/Bc mice on both high Mg^2+^ diets maintained similar basal plasma Mg^2+^ values. However, as predicted, when mice were placed on the control Mg^2+^ casein diet, average plasma Mg^2+^ levels dropped significantly after 15 days (0.56 ± .01) and slightly further after 60 days (0.49 ± .01), although the additional decrease was not itself statistically significant.

##### 3.4.1.2 LM/Bc urine Mg^2+^

The results for urinary Mg^2+^ excretion on the experimental casein diets at time points corresponding to plasma Mg^2+^ quantifications are shown in Figure 3B. After 15 days on the high Mg^2+^ casein diet, urinary Mg^2+^ excretion (µmol/24 h ± SEM) in LM/Bc mice was *highly variable*, and on average, significantly higher (87.90 ± 15.50) than on the high Mg^2+^ soy diet (41.07 ± 2.16). The .3% Mg oxide added to the purified casein diet is predicted to be more bioavailable and more readily absorbed from the gut than the .3% Mg in the soy natural ingredient diet due to chelation of Mg^2+^ by phytate in plant sources (Gibson et al., 2018; Marolt et al., 2020). Monogastric animals do not have phytases in their digestive tract to break down phytic acid and release bound Mg^2+^ for absorption, so bio-accessibility in the soy diet may be <.3%. This could explain the higher urinary Mg^2+^ output for mice on the high Mg^2+^ casein diet vs the high Mg^2+^ soy diet; despite that disparity in urine output between the 2 high Mg^2+^ diets, basal plasma Mg^2+^ levels remained comparable. However, when mice were placed on the control Mg^2+^ casein diet, urine Mg^2+^ output declined significantly after 15 days (20.19 ± 2.56) and 60 days (14.26 ± 3.62).

#### 3.4.2 Impact of reduced dietary Mg^2+^ intake on plasma and urine Mg^2+^ in SWV mice

To determine the role of deficient maternal dietary Mg^2+^ intake on DTG-NTD susceptibility in the SWV strain, newly weaned female SWV mice (from lactating dams maintained on the natural ingredient high Mg^2+^ soy diet) were placed on the Mg^2+^-deficient casein diet. Blood and urine samples were collected prior to the start of the experimental diet and again after 2 weeks (15 days) on the diet for ICP-MS determination of cations.

##### 3.4.2.1 SWV plasma Mg^2+^

The results for diet effect (food matrix and Mg^2+^ concentration) on SWV plasma Mg^2+^ levels are shown in Figure 3C. Compared to plasma Mg^2+^ levels (mmol/L ± SEM) in SWV mice on the high Mg^2+^ soy diet (1.05 ± 0.08), average plasma Mg^2+^ decreased significantly after 15 days on the high Mg^2+^ casein diet (0.77 ± 0.07), although the reason(s) for this are not clear and may involve compensatory mechanisms such as release of parathyroid hormone that shift the homeostatic ‘set point’ in response to excess dietary Mg^2+^.

*Mg* ^*2+*^ *-Deficient Diet:* Plasma Mg^2+^ declined drastically in SWV after 15 days on the Mg^2+^-deficient casein diet (0.39 ± 0.11) to levels comparable with those observed in LM/Bc mice maintained on control Mg^2+^ casein diet for 60 days (0.49 ± .01).

##### 3.4.2.2 SWV urine Mg^2+^

The impact of changing from high Mg^2+^ soy to high or deficient Mg^2+^ casein diets on urinary Mg^2+^ excretion in SWV mice is shown in Figure 3D. After 15 days on the high Mg^2+^ casein diet, urinary Mg^2+^ excretion (µmol/24 h ± SEM) in SWV mice was *highly variable*, and significantly higher (138.52 ± 23.30) than on the high Mg^2+^ soy diet (22.82 ± 4.37), the same trend that was observed for the LM/Bc strain. Increased Mg^2+^ bio-accessibility and elevated urinary Mg^2+^ output after 15 days on the high Mg^2+^ casein diet might explain the corresponding decline in plasma Mg^2+^ in SWV mice.

*Mg* ^*2+*^-*Deficient Diet:* After 15 days on the Mg^2+^-deficient casein diet, urinary Mg^2+^ excretion decreased significantly (3.38 ± 1.25) relative to the high Mg^2+^ casein diet, indicative of renal conservation and reabsorption of Mg^2+^ in the SWV strain to compensate for the severe restriction in dietary Mg^2+^ intake.

### 3.5 Impact of reduced maternal dietary Mg^2+^ intake on susceptibility to DTG-NTDs

#### 3.5.1 LM/Bc: High Mg^2+^ casein diet x DTG exposure

**FIGURE 4:**
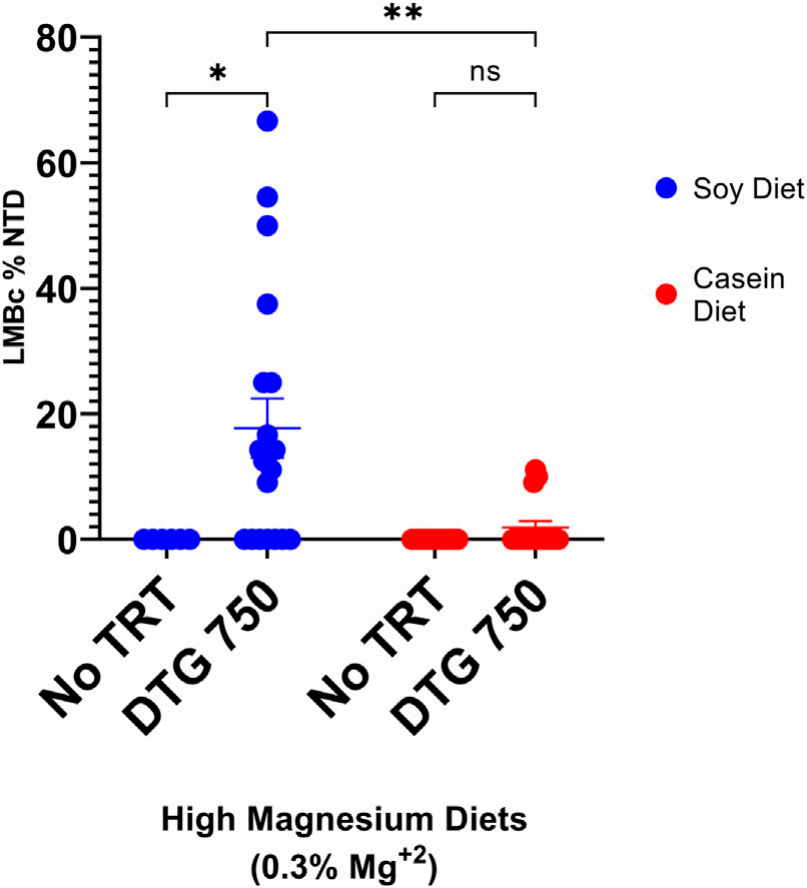
NTDs IN LM/Bc MICE ON HIGH Mg^2+^ DIETS (SOY vs CASEIN) x DTG750. LM/Bc mice were placed on either soy (blue dots) or casein (red dots) high Mg^2+^ (0.3%) diet for a minimum of 4 weeks prior to timed matings. Susceptibility to NTDs following administration of DTG (750 mg/kg/d oral gavage from GD 0.5—GD 9.5) was compared within each diet to a diet only control group (no treatment). Comparisons were also made between the two high Mg^2+^ (soy vs casein) diets in dams treated with DTG750. [An additional n = 6 dam on each diet received vehicle to confirm previous results that vehicle treatment alone did not cause NTDs; no NTDs were found in any of these litters (data not shown on graph)]. The dam was considered the experimental unit and comparisons shown are between litter means (average % of embryos in each litter that had an NTD). A litter was considered affected if at least 1 embryo presented with exencephaly (NTD). The % DTG-NTD-affected litters (63% soy vs 19% casein) and mean % affected embryos/litter (shown on graph) were both higher on the high Mg^2+^ soy diet compared to the high Mg^2+^ casein diet. (*p < 0.05, **p < 0.01)

No NTDs were observed in LM/Bc mice (n = 12 litters, 89 implants) on the high Mg^2+^ casein diet only. However, in LM/Bc dams on the high Mg^2+^ casein diet who were treated with DTG750 90 implants total), 3/16 litters (19% NTD-affected litters; mean rate 2% NTDs/litter) each had n = 1 exencephalic embryo. This was significantly fewer NTD-affected litters compared to LM/Bc dams treated with DTG750 that were maintained on the high Mg^2+^ soy diet (63% NTD-affected litters; mean rate 18% NTDs/litter) (Figure 4). The higher incidence of DTG-NTD affected litters and mean % affected embryos/litter on the high Mg^2+^ soy diet is likely due to phytate chelation of Mg^2+^ in the plant-based diet and therefore less bio-accessibility of Mg^2+^ in the soy-vs. the casein-based diet.

#### 3.5.2 LM/Bc: control Mg^2+^ casein diet x DTG exposure

**FIGURE 5:**
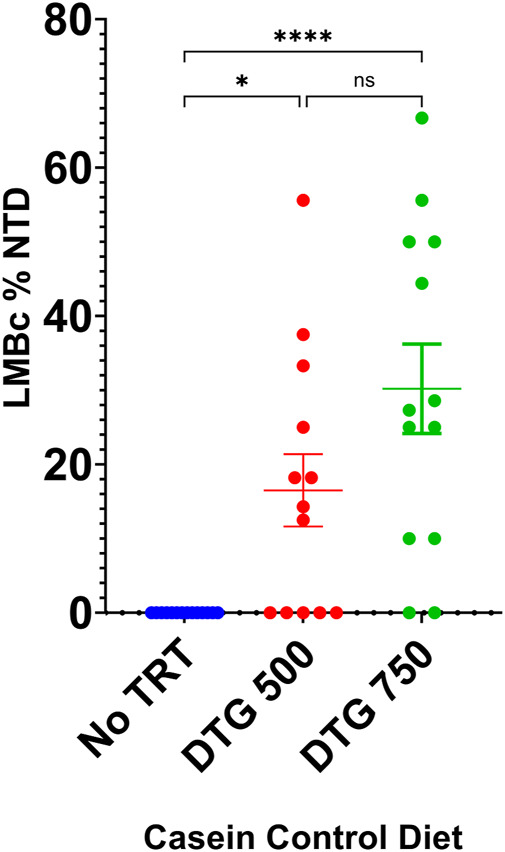
DTG-NTD dose-response in LM/Bc mice on casein control Mg^2+^ diet. LM/Bc mice were placed on the control Mg^2+^ (0.05%) casein diet for a minimum of 4 weeks prior to timed matings. Pregnant mice on diet only control (no treatment) were compared to mice treated with either DTG500 or DTG750 (oral gavage from GD 0.5—GD 9.5) and litters examined for NTDs. [No NTDs were observed in litters from an additional n = 6 dam on control Mg^2+^ diet treated with vehicle (data not shown)]. Comparisons shown are between litter means (average % of embryos in each litter that had an NTD). No NTDs were observed in diet only control litters (blue dots). The % DTG-NTD-affected litters (62% for DTG500 and 85% for DTG750) and mean % affected embryos/litter (shown on graph) were both significantly higher than the diet only control group, but DTG500 (red dots) and DTG750 litter means (green dots) were not significantly different from each other. (*p < 0.05, ****p < 0.0001).

LM/Bc mice placed on the control Mg^2+^ casein diet for a minimum of 4 weeks continued to appear healthy and were placed into timed matings. Plugged females were randomly placed into one of 4 experimental groups: 1) diet-only control (no treatment); 2) vehicle; 3) DTG500; or 4) DTG750. No NTDs were observed in litters from LM/Bc mice on the control Mg^2+^ casein diet only (n = 14) or in litters from dams on the control Mg^2+^ casein diet who were treated with vehicle (n = 6). However, exencephalic embryos were observed when LM/Bc dams were administered either DTG750 (n = 13 litters) or DTG500 (n = 13 litters). In DTG750-treated dams, 11/13 litters (85%) had 1 or more exencephalic embryos (total of 33 NTDs/105 implants), ranging from 1 to 6 per litter. In DTG500-treated dams, 8/13 litters (62%) had 1 or more exencephalic embryos (total of 19 NTDs/116 implants), ranging from 1 to 5/litter. These results are depicted graphically in Figure 5. Fewer litters were affected with NTDs when exposed to DTG500 compared to DTG750; however, because of the within-litter variability in number of NTDs, analysis of litter means did not detect a statistically significant difference between dosages. Both DTG dosages were statistically significant compared to the diet-only control. Determination of the NOAEL and dose-response relationship will require testing lower doses of DTG in LM/Bc mice on the control Mg^2+^ casein diet. The present results demonstrate that LM/Bc mice are susceptible to NTDs at a lower DTG dose when dietary Mg^2+^ intake is reduced and hence maternal plasma Mg^2+^ levels are lower at the time of conception and start of DTG treatment.

#### 3.5.3 SWV: Deficient Mg^2+^ casein diet x DTG exposure

**FIGURE 6:**
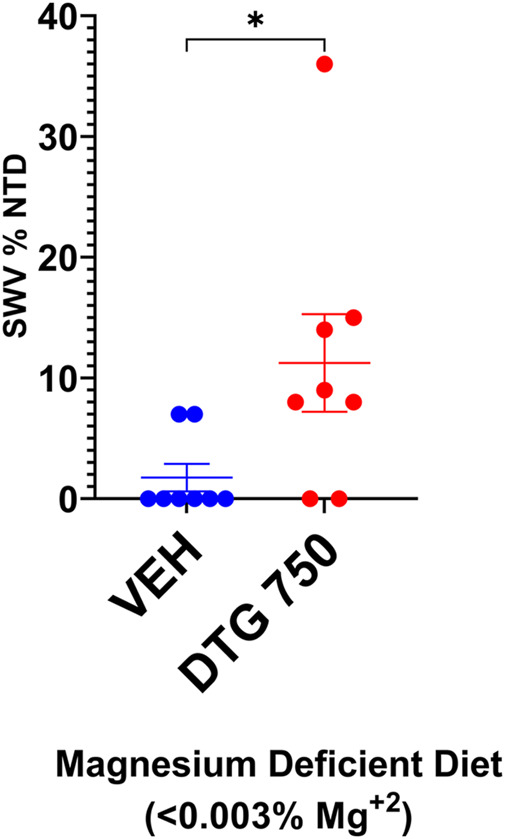
NTDs IN SWV MICE ON Mg2+ DEFICIENT DIET x DTG750. SWV mice were placed on Mg^2+^ deficient (<0.003%) casein diet for a minimum of 4 weeks prior to timed matings. Pregnant mice received either no treatment (diet-only control) or DTG750 (oral gavage from GD 0.5—GD 9.5). NTDs were observed in 25% of diet only control litters and 75% of litters treated with DTG750. The mean % affected embryos/litter (shown on graph) was significantly higher in the DTG750-treated group (red dots) than in the diet only control group (red dots). (*p* < .05).

After a minimum of 4 weeks on the Mg^2+^-deficient casein diet, SWV females appeared in good health and were placed in timed matings. Plugged females received either no treatment (diet-only control) or the Mg^2+^-deficient casein diet + DTG750 to test whether severely reduced dietary intake of Mg^2+^ would increase susceptibility to DTG-NTDs (n = 8 females/treatment group). The results are shown in Figure 6. NTDs were observed in 2/8 (25%) litters (n = 1 exencephalic pup in each litter) among mice on the Mg^2+^-deficient casein diet only, demonstrating that maternal dietary Mg^2+^ deficiency alone compromises neural tube closure in SWV mice. In SWV dams on the Mg^2+^-deficient casein diet + DTG750, NTDs were observed in 6/8 (75%) litters (total of 11 NTDs, ranging from 1-4 per affected litter). The results demonstrate a combined diet x drug effect and increased susceptibility to DTG- NTDs (in an otherwise relatively resistant strain) when dietary Mg^2+^ intake is reduced and hence maternal plasma Mg^2+^ levels are lower at the time of conception and start of DTG treatment.

### 3.6 Analysis of variants in magnesium homeostasis pathway genes

A diet x drug effect and increased susceptibility to DTG-NTDs was observed in both LM/Bc and SWV mice when maternal plasma Mg^2+^ levels were lower prior to the onset of drug treatment due to reduced dietary Mg^2+^ intake. However, the LM/Bc strain was more susceptible to DTG-NTDs than the SWV strain when dietary Mg^2+^ levels were sufficient, suggesting an underlying genetic predisposition. To identify genetic variants that might contribute to perturbations in Mg^2+^ homeostasis and hence the increased susceptibility of the LM/Bc strain to DTG-NTDs, we reanalyzed a whole-exome sequencing data set previously generated from these 2 strains.

The summary statistics for the analyses of all variants in the SWV and LM/Bc mouse strains compared to a C57BL/6J reference are shown in Supplementary Table S2. All SNVs and insertions/deletions (indels) found in the DTG-NTD susceptible LM/Bc strain are shown in Supplementary Table S3; Supplementary Table S4. No variants in *Ugt1a1*, *Ugt1a3*, *Ugt1a9*, or *Cyp3a4* (genes involved in DTG metabolism) were found that were unique to LM/Bc (validated by Transnetyx, Inc, Cordova, TN). One high-risk variant in a known magnesium homeostasis gene was identified in LM/Bc but not in SWV–a heterozygous frameshifting variant in *Hnf1b* (Supplementary Table S4). However, Sanger validation (Supplementary Figure S1) showed that this was likely a false positive call in a difficult-to-sequence region of the genome.

We also looked for variants unique to LM/Bc with a predicted deleterious effect (SIFT) that are intolerant to mutation in humans (pLI >0.9 applied to putative LoF variants or a missense z > 3 applied to missense variants; gnomAD, Broad Institute). This list included 17 SNVs in 14 genes (*Nck2*, *Eef2*, *Gck*, *Smurf2*, *Scn8a*, *Vps52*, *Psmc3*, *Camta1*, *Rbpj*, *Ran*, *Cdk8*, *Kcnn1*, *Ano8*, and *Dnmt1*) shown in Supplementary Table S3. Six of these variants have risen to a homozygous state in the LM/Bc strain.

*Identification of Unique Fam111a Variants in LM/Bc:* For missense variants, we further filtered for those with a predicted deleterious effect as determined by SIFT, which identified a collection of 9 Sanger-verified SNVs in the gene *Fam111a*. LM/Bc mice are homozygous for all 9 of the predicted deleterious variants. Thirty-seven additional benign SNVs were also found in this gene in the LM/Bc strain but not in SWV or in the C57BL/6J reference genome. Three of the same benign SNVs were found in the background C3H/HeJ strain, but none were predicted to be protein disruptive. All benign variants falling within the Sanger amplicon containing the 9 deleterious variants were also validated. The results are shown in Supplementary Figure S2. SNVs are shown in bold red font. In PCR reactions, the Fam111a_F primer was used to amplify all samples, with Fam111a_R for SWV and Fam111a_R2 for LM/Bc. Sanger sequencing was performed using the Fam111a_F primer at Genewiz/Azenta Life Sciences and consensus calling was manually checked from chromatograph traces using GeneSnap Viewer v5.3.2. Additional tail samples from mice used in these experiments and mice randomly selected from the breeding colony were screened by Transnetyx, Inc. (Cordova, TN) Automated Genotyping Services to confirm that the LM/Bc mice currently in-house remain homozygous for the 9 deleterious variants identified in *Fam111a*.

The human FAM111A protein (NP001299838) is a 611 amino acid serine protease that contains a conserved catalytic triad [reviewed in Welter and Machida, 2022] with chymotrypsin-like specificity at the C- terminus and an autocleavage site between Phe334 and Gly335 (Hoffmann et al., 2020; Kojima et al., 2020). Its known functions include 1) interacting directly with PCNA and nascent chromatin to load PCNA at the DNA replication fork; 2) removing protein obstacles at the replication fork to prevent stalling and promote DNA synthesis; and 3) acting as a host restriction factor in antiviral defense (Kojima et al., 2020; Nie et al., 2021) against SV40 polyomavirus (Fine et al., 2012; Tarnita et al., 2018), orthopoxvirus (Panda et al., 2017), and Zika virus (Ren et al., 2021). Depletion of FAM111A in virus-infected cells disrupts host cell nuclear pore proteins and barrier function, leading to increased formation of viral replication centers (Hoffmann et al., 2020; Nie et al., 2021; Welter and Machida, 2022).

**FIGURE 7:**
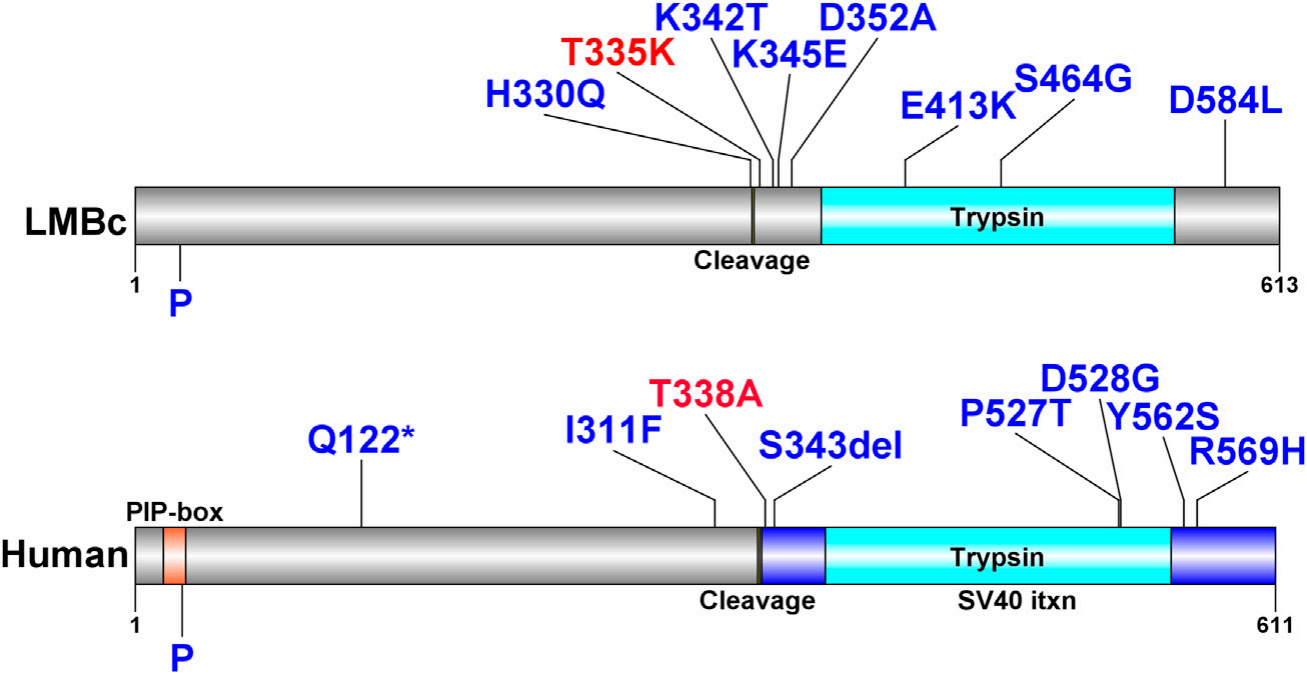
FAM111A protein plots with variants in humans and LM/Bc mice. FAM111A protein plots in humans and LM/Bc mice. Protein plots show locations of known protein domains (colored and annotated regions). Variants predicted to be deleterious found only in the LM/Bc strain are shown (top) as are likely pathogenic and pathogenic variants found in humans in ClinVar (bottom). The variants highlighted in red are aligned between mouse (T335) and human (T338). P: phosphorylation site.

The positions of the deleterious missense variants identified in *Fam111a* in LM/Bc mice and corresponding amino acid changes in the protein are shown in Table 4. The locations of known human variants in *FAM111A* and the 9 predicted deleterious variants in *Fam111a* in the LM/Bc mouse strain are shown in Figure 7. Five of the 9 LM/Bc variants are within or near the autocleavage site. Two are within the catalytic domain and alter the charge or polarity of an amino acid (aa 413, 464). The remaining 2 variants near the C-terminus are alternate variants at the same location; both result in a charge-altering amino acid change (aa 584).

**TABLE 4:** Predicted deleterious SNVs in LM/Bc Fam111a. Whole exome sequencing data generated for the inbred LM/Bc and SWV strains was analyzed to identify potential variants in genes involved in Mg^2+^ homeostasis. The positions of the predicted deleterious missense variants identified in *Fam111a* that are unique to LM/Bc mice are shown along with the corresponding amino acid change in the protein. The T335K variant in LM/Bc mice corresponds to the T338A variant in humans that is associated with the osteocraniostenosis (OCS) phenotype (OMIM# 602361).

SNV location (LM/Bc)	Position and AA change (mouse)	PROTEIN domain (mouse)	Side chain charge	CORRESPONDING human AA *conserved	Condition (human variant)
rs52218749	330 His → Gln	Autocleavage site	Basic → Polar	Thr 333	
rs49986153	335 Thr → Lys	Near autocleavage site	Polar → Basic	Thr 338*	OCS (T338A)
rs46660569	342 Lys → Thr		Basic → Polar	Lys 345*	
rs240389514	345 Lys → Glu		Basic → Acidic	Lys 348*	
rs47274130	352 Asp → Ala		Acidic → Nonpolar	Asp 355*	
rs263904583	413 Glu → Lys	Catalytic domain	Acidic → Basic	Glu 415*	
rs258735034	464 Ser → Gly	Catalytic domain	Polar → Nonpolar	Ser 466*	
rs263418225	584 Asp → His		Acidic → Basic	Leu 582	
rs225390403	584 Asp → Val		Acidic → Nonpolar	Leu 582	

In humans, most of the heterozygous *de novo* missense variants that have been identified result in ‘gain of function’ and hyperactivity of the protease (Hoffmann et al., 2020). Mutations in human *FAM111A* are associated with Kenny-Caffey Syndrome, type 2 (KCS2, OMIM #127000), in which patients exhibit increased bone density, growth retardation, macrocephaly, facial dysmorphism, hypoparathyroidism, and electrolyte imbalances, including hypocalcemia and hypomagnesemia (Isojima et al., 2014; Abraham et al., 2017) or Gracile Bone Dysplasia/Osteocraniostenosis (GCLEB/OCS, OMIM #602361), characterized by skeletal/craniofacial malformations, and perinatal lethality (Unger et al., 2013; Muller et al., 2021; Rosato et al., 2022). In LM/Bc mice, only 1/9 of the identified deleterious missense variants correspond to a known human variant: the T335K variant in LM/Bc near the autocleavage site corresponds to the T338A variant in humans that have an OCS phenotype. Although we have not examined skeletal preparations, we do not grossly observe the severe skeletal malformation phenotype described for OCS in humans. We do, however, frequently observe microphthalmia (often unilateral) in the colony, and the occasional birth of small (runted) pups that fail to thrive, consistent with the ocular malformation and perinatal lethality phenotype described for humans with OCS. Based on results in the current study, LM/Bc mice exhibit hypomagnesemia and hypocalcemia, consistent with the human phenotype described for KCS2. Human variants in *FAM111A* are associated with hypomagnesemia and renal Mg^2+^ wasting; this phenotype in LM/Bc mice is thought to contribute to increased DTG-NTD susceptibility. Although several *Fam111a* deleterious variants unique to the LM/Bc strain were identified in this study, the significance of these mutations with respect to phenotype remains to be determined.

## 4 Discussion

Our results demonstrate the multifactorial etiology of NTDs and the complex gene-nutrient-environment interactions that contribute to risk for this type of congenital malformation. In addition, the data supports a role for maternal Mg^2+^ status as an underlying risk factor for DTG-NTDs. Magnesium is an important micronutrient, but its critical role in pregnancy is often overlooked. Genetic variants, inadequate dietary intake, and various disease conditions and/or medications can lead to chronic latent hypomagnesemia. In individuals who have one or more of these risk factors and subclinical hypomagnesemia, starting DTG in the months before conception may tip the balance towards an NTD affected pregnancy.

Numerous genetic variants predispose to hypomagnesemia. To test our hypothesis, we selected mouse strains on diverse genetic backgrounds with a range of inherently different basal plasma Mg^2+^ levels. LM/Bc mice had low basal plasma Mg^2+^ levels and elevated urinary Mg^2+^ excretion, but the underlying genetic cause was unknown. The LM/Bc strain was the most susceptible to DTG-NTDs, whereas C57BL/6J mice (highest basal plasma Mg^2+^) proved to be the most resistant. Our WES study identified several unique variants in the *Fam111a* gene in LM/Bc mice. In humans, *FAM111A* variants are associated with hypomagnesemia and renal Mg^2+^ wasting, consistent with the phenotype observed in LM/Bc mice. Pre-existing perturbations in Mg^2+^ homeostasis appear to be an underlying risk factor for DTG-NTDs in LM/Bc mice. However, it will be important to corroborate these findings by testing other mouse models with mutations in Mg^2+^ homeostasis pathway genes.

Our results also underscore the importance of adequate Mg^2+^ intake during pregnancy. Dietary intake of this micronutrient impacted both maternal plasma Mg^2+^ levels and susceptibility to DTG-NTDs. The food matrix also affected Mg^2+^ bio-accessibility, homeostasis, and susceptibility to DTG-NTDs. In LM/Bc dams maintained on a high Mg^2+^ (soy or casein) diet, none of the vehicle-treated litters were affected with an NTD. However, when exposed to DTG750, 63.2% of the LM/Bc litters on high Mg^2+^ soy diet had 1 or more NTD, while only 19% of litters were NTD-affected on the high Mg^2+^ casein diet. This was an unexpected difference, but can likely be attributed, at least in part, to phytate chelation of Mg^2+^ in the plant (soy)-based diet, which makes the 0.3% Mg^2+^ less bio-accessible (Gibson et al., 2018; Marolt et al., 2020; Sun et al., 2021). Notably, nutritionists, food scientists, and plant geneticists are aware of this issue, and are working on strategies to reduce phytic acid in cereal grains, legumes, and nuts to help alleviate this type of micronutrient deficiency in countries that rely heavily on plants as the major food source (Gupta et al., 2015; Vashishth et al., 2017).

Insufficient dietary Mg^2+^ intake and subclinical hypomagnesemia is a prevalent condition in humans worldwide, even in developed countries (Dalton et al., 2016). When LM/Bc mice were placed on casein diet with Mg^2+^ reduced to the NRC recommended control level (0.05%), plasma Mg^2+^ levels dropped significantly. Despite this, mice appeared healthy, and embryos developed normally in vehicle-treated dams. However, when LM/Bc dams on this diet were treated with DTG750, 85% of litters had 1 or more pups with exencephaly, and when given a lower dose of DTG500, 62% of litters were NTD-affected. SWV female mice placed on a severely restricted Mg^2+^ deficient diet became hypomagnesemic prior to conception, and 25% of litters were NTD-affected in the diet only control group. The proportion of NTD-affected litters increased to 75% when SWV dams were treated with DTG750. In addition to the data presented, offspring from 2 additional SWV litters on Mg^2+^ deficient diet were examined at GD 12.5. In both, the entire litter was in the process of being resorbed, suggesting that severe dietary Mg^2+^ deficiency is incompatible with fetal development and term pregnancy. Moving forward, to reproduce a more likely scenario in humans, it would be preferable to test DTG-NTD susceptibility in mice on a ‘low’ Mg^2+^ diet that is compatible with a healthy pregnancy, rather than one so severely Mg^2+^ deficient that diet alone causes NTDs and fetal death.

Another potential hidden dietary risk factor for altered Mg^2+^ homeostasis and NTDs is exposure to the mycotoxin fumonisin B1 (FB1). FB1 is a common contaminant found in maize-based foods worldwide, including many countries in Africa where rollout of DTG has been widespread (Yli-Mattila and Sundheim, 2022; Alberts et al., 2019; Omotayo et al., 2019). FB1 inhibits the enzyme ceramide synthase in *de novo* sphingolipid biosynthesis, causing accumulation of sphingoid bases (sphingosine and sphinganine) in human and mouse blood (Riley et al., 2015a; Riley et al., 2015b), and in mouse placentas and embryos (Gelineau-van Waes et al., 2005). Sphingosine is a potent inhibitor of the magnesium transporter TRPM7 (Qin et al., 2013). Subclinical hypomagnesemia in LM/Bc mice may therefore also have been a predisposing risk factor for susceptibility to FB1-NTDs (Gelineau-van Waes et al., 2012). In cultured mouse embryos, the incidence of FB1^−^NTDs could be reduced by adding folic acid to the culture medium (Sadler et al., 2002). Daily folic acid supplementation was also partially protective against FB1-NTDs in pregnant LM/Bc dams maintained on a folate-sufficient diet (Gelineau-van Waes et al., 2005). The diets used for the DTG studies were folate replete, but it will be important to determine if additional maternal folic acid supplementation is effective at reducing risk for DTG-NTDs.

In addition to dietary Mg^2+^ insufficiency, genetic variants that alter Mg^2+^ homeostasis represent known risk factors for NTDs. In the early embryo, magnesium plays an important role in cell migration and tissue morphogenesis through regulation of noncanonical Wnt/planar cell polarity signaling (Komiya et al., 2014; Komiya et al., 2017; Runnels and Komiya, 2020). Mg^2+^ transport via TRPM7 is important for mediolateral intercalation, cell polarity, adhesion, and axis elongation during early gastrulation, while Mg^2+^ transport via TRPM6 is required for radial intercalation during neural tube closure (Komiya et al., 2017; Runnels and Komiya, 2020). In mice, homozygous deletion of *Trpm7* is embryonic lethal, and *Trpm6* knockout mutants exhibit exencephaly and/or spina bifida occulta (Walder et al., 2009; Woudenberg-Vrenken et al., 2011; Jin et al., 2008). In humans, mutations in *TRPM6* cause familial hypomagnesemia with secondary hypocalcemia (OMIM #607009) (Walder et al., 2002; Schlingmann et al., 2002), and a *TRPM6* variant has been associated with meningomyelocele (Sarac et al., 2016).

Our WES analysis of inbred LM/Bc and SWV mouse strains and comparison to the C57BL/6J reference genome identified 9 unique homozygous *de novo* missense variants in *Fam111a* in the LM/Bc strain. In LM/Bc mice, several of the predicted deleterious *Fam111a* variants are near the autocleavage site (Figure 7), and the T335K variant corresponds to the pathogenic T338A variant in humans. Human GOF variants in *FAM111A* are associated with hypomagnesemia and hypermagnesuria (Viering et al., 2017), but exactly how FAM111a is involved in regulation of renal Mg^2+^ transport is not clear. *Fam111a* knockout (KO) mice on a C57BL/6N genetic background maintained on standard rodent chow containing 0.22% Mg^2+^ are viable, and display no overt phenotype (Ilenwabor et al., 2022). The KO mice have normal bone, kidney, and parathyroid morphology, and normal serum and urine Mg^2+^, Ca^2+^ and phosphate levels. Based on the lack of electrolyte abnormalities in *Fam111a* KO mice, and the fact that missense variants in humans lead to GOF, hypocalcemia, and hypomagnesemia, we predict that one or more of the missense *Fam111a* variants identified in LM/Bc mice also result in GOF, although this remains to be determined.

In humans, disease-causing mutations in *FAM111A* result in a phenotype that resembles activating mutations in the G protein-coupled Calcium Sensing Receptor (CASR). It has been proposed that FAM111A is a potential modifier of CASR (Tan et al., 2021), and that GOF in FAM111A protease activity may result in constitutive activation of CASR by preventing its internalization and downregulation (Pi et al., 2005; Mos et al., 2019). CASR is expressed in the parathyroid glands, intestines, and kidneys, and plays a pivotal role in homeostatic regulation of Ca^2+^ and Mg^2+^ (Chavez-Abiega et al., 2020). Heterozygous activating mutations in *CASR* cause Autosomal Dominant Hypocalcemia, type 1 (ADH1), or Bartter’s Syndrome, type V (both OMIM# 601198). ADH1 is characterized by low serum Ca^2+^ and hypercalciuria; Bartter’s Syndrome is more severe, and patients exhibit defective renal sodium chloride transport and increased urinary sodium excretion, which alters the electrochemical gradient necessary for Mg^2+^ and Ca^2+^ reabsorption. This leads to renal Mg^2+^ wasting, hypocalcemia, and hypomagnesemia (consistent with the phenotype observed in LM/Bc mice). In the thick ascending loop of Henley, CASR regulates paracellular transport of Ca^2+^ and Mg^2+^ through the cation-selective pore formed by claudin-16 (CLDN16) and claudin-19 (CLDN19) (Gunzel and Yu, 2009; Downie and Alexander, 2022). Human *CLDN16* and *CLDN19* variants are associated with familial hypomagnesemia with hypercalciuria (FHHNC; OMIN 248250), and a missense variant in *CLDN19* has been associated with NTDs (Baumholtz et al., 2020).

GOF mutations in *Fam111a* that in turn result in activation of CASR present a plausible link between altered Mg^2+^ homeostasis and mechanisms that contribute to failure of neural tube closure. In colon cancer cells, CASR activation stimulates secretion of WNT5A in the noncanonical Wnt signaling pathway (MacLeod et al., 2007). WNT5A regulates epithelial-mesenchymal transformation and migration of axial and paraxial mesodermal precursors in early embryos (Andre et al., 2015), and is important for planar cell polarity (PCP), convergent extension, anterior-posterior axis elongation during gastrulation, and proper patterning of the notochord, somites, and neural tube (Andre et al., 2015). Loss of *Wnt5a* (in KO mice) results in axial shortening, limb truncations (Yamaguchi et al., 1999), and NTDs (Qian et al., 2007). In the developing embryo, noncanonical WNT5a interacts with its co-receptor LRP5/6 to inhibit canonical β-catenin signaling (Andersson et al., 2010; Bryja et al., 2009; Topol et al., 2003). The balance between these two signaling pathways is important because both loss and gain-of-function in noncanonical Wnt signaling can result in failure of neural tube closure (Allache et al., 2014; Allache et al., 2015; Shi, 2022; Shariatmadari et al., 2005). It is therefore interesting that in the hESC 3D morphogenesis model, *WNT5A* expression was upregulated 5 days after exposure to DTG (Kirkwood-Johnson et al., 2021). The authors also observed upregulation in several genes in the retinoic acid (RA) pathway. RA suppresses canonical Wnt/β-catenin in ESC and activates non-canonical WNT5A (Osei-Sarfo and Gudas, 2014), lending further support to potential involvement of this pathway in DTG-NTDs.

**FIGURE 8:**
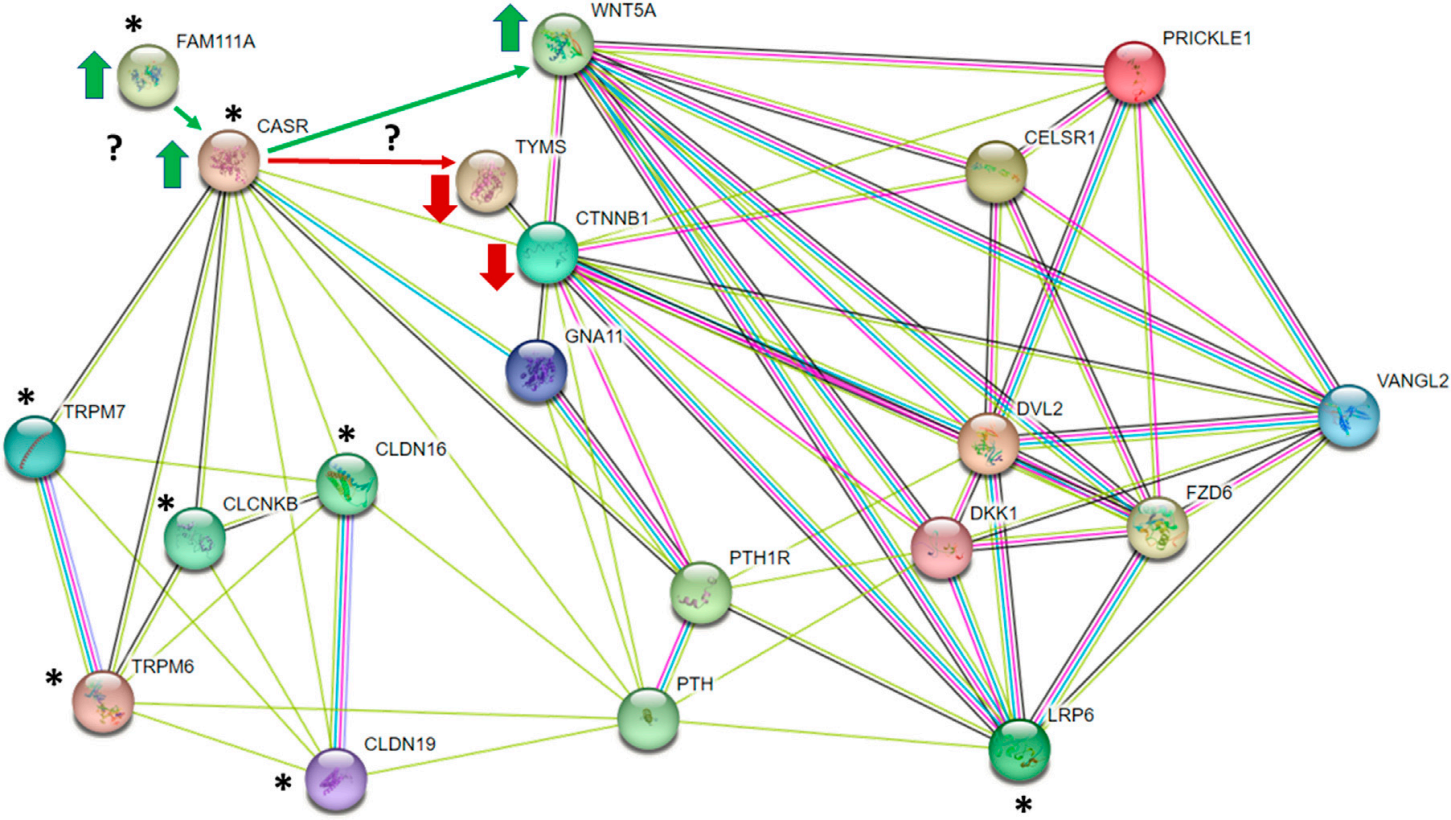
STRING diagram illustrating potential connections between gain-of-function mutations in *Fam111a* and activation of CASR that might in turn impact the balance of WNT5A/planar cell polarity and β-catenin (CTNNB1) signaling pathways and/or inhibit thymidylate synthase (TYMS). * represent proteins involved in regulation of magnesium homeostasis.

Another possible connection between GOF mutations in *Fam111a*, activation of CASR and DTG-NTDs ties into the folate pathway. In human colon cancer cells, CASR activation downregulates expression of thymidylate synthase (TYMS), a key enzyme involved in DNA synthesis (Liu et al., 2010; Liu et al., 2011). TYMS catalyzes the conversion of dUMP and 5, 10-methylenetetrahydrofolate to dTMP and 7,8-dihydrofolate. Activation of CASR and inhibition of TYMS leads to reduced dTMP biosynthesis, accumulation of dUMP, and misincorporation of uracil in DNA, a scenario associated with NTDs in humans and mice (Dunlevy et al., 2007; Beaudin et al., 2011). Inhibition of TYMS by the anti-folate drugs raltitrexed or 5-fluorouracil cause NTDs in mice (Dong et al., 2015; Wang et al., 2018) and human TYMS polymorphisms are associated with NTD risk (Volcik et al., 2003). Collectively, GOF mutations in *Fam111a* that in turn might result in activation of CASR, secretion of WNT5A, and inhibition of both β-catenin and TYMS represent plausible links between perturbations in magnesium homeostasis and increased susceptibility to DTG-NTDs in LM/Bc mice that warrant future investigation. The connections between these and other signaling molecules with known roles in neural tube closure (CELSR1, VANGL2, PRICKLE1, DVL2) are illustrated in the STRING diagram in Figure 8.

In addition to dietary insufficiency and/or genetic causes, medications and disease conditions can lead to hypomagnesemia, including alcoholism, coeliac disease, diarrhea, and chronic kidney disease (CKD) (Katopodis et al., 2020; Rodelo-Haad et al., 2020), a prevalent comorbidity for patients living with HIV (Wearne et al., 2020). Medications that interfere with transporters involved in renal Mg^2+^ reabsorption, include proton pump inhibitors and diuretics, among others (Bosman et al., 2021; Atsmon and Dolev, 2005). Of particular importance in HIV/AIDS patients, aminoglycoside antibiotics used to combat tuberculosis and other bacterial infections, or the antifungal agent amphotericin B can cause nephrotoxicity, renal Mg^2+^ wasting, and hypomagnesemia (Barton et al., 1984). Pentamidine, used to treat pneumocystic pneumonia, and parasitic infections, can also cause acute kidney injury, renal Mg^2+^ loss, hypocalcemia, and hypomagnesemia. Foscarnet is used to treat Cytomegalovirus retinitis, another opportunistic infection in HIV/AIDS patients (Gearhart and Sorg, 1993; Huycke et al., 2000). Foscarnet chelates divalent cations leading to decreased plasma concentrations of Ca^2+^ and Mg^2+^ (Jacobson et al., 1991). Monitoring plasma Mg^2+^ levels when on these medications should therefore be included in the treatment plan, particularly in women of childbearing age.

Perturbations in Mg^2+^ homeostasis and hypomagnesemia can lead to various metabolic disorders, including altered glucose and lipid metabolism, insulin resistance, and T2DM (Feng et al., 2020; Wells, 2008; Pelczynska et al., 2022), an established risk factor for NTDs (Lupo et al., 2012). Amniotic fluid Mg^2+^ levels are low in pregnant women with T2DM (Mimouni et al., 1987), which could impair normal embryonic development. Recent reports indicate that the use of INSTI-based ART regimens, including DTG, lead to weight gain and increased risk for T2DM (McCann et al., 2021; Bailin and Koethe, 2023). Patients present with hyperglycemia between 2–5 months after starting DTG therapy (Lamorde et al., 2020). If, in fact, hyperglycemia and increased risk for T2DM is associated with DTG-induced perturbations in Mg^2+^ homeostasis, the gradual onset (2–5 months) to appearance of clinical symptoms supports the hypothesis that DTG might result in a slow decline in plasma Mg^2+^ over time, although this remains to be verified in the clinical setting. The timing would also agree with the observation that NTDs were only observed in women who started DTG therapy 3 months prior to conception. Similarly, NTDs were only observed in animal models when DTG was started at the time of conception.

Our studies focused on the role of perturbations in maternal Mg^2+^ homeostasis as an underlying risk factor for DTG-NTDs, but it will also be critical to examine the impact of DTG on Mg^2+^ dynamics at the level of the placenta and embryo. The major Mg^2+^ transporters involved in intestinal uptake and renal reabsorption in the dam, TRPM6 and TRPM7, are also expressed in visceral yolk sac endoderm, extraembryonic chorion, trophoblast cells (De Clercq et al., 2021) and the early embryo (Komiya et al., 2014; Komiya et al., 2017; Runnels and Komiya, 2020). During neurulation, nutrients reach the embryo by histotrophic mechanisms that involve the visceral yolk sac [reviewed in Zohn and Sarkar, 2010]. In humans, the extraembryonic coelomic fluid acts as a nutrient reservoir for the developing embryo, and in the first trimester, magnesium (and folate) are higher in coelomic fluid than in amniotic fluid (Wathen et al., 1995; Campbell et al., 1993). It is unknown to what extent unbound DTG reaches these tissues and fluids and how this affects Mg^2+^availability to the developing embryo. Establishment of hemotrophic nutrition and exchange of nutrients (and drugs) between maternal and fetal circulation does not occur until ∼10–12 weeks gestation in humans, long after the neural tube is closed. Transplacental transfer of DTG has been demonstrated in women during the second and third trimester (Mulligan et al., 2018; Bollen et al., 2021; Waitt et al., 2019) and *ex vivo* in a term human placenta cotyledon perfusion model (Schalkwijk et al., 2016; Mandelbrot et al., 2019). However, no information is available in either mice or humans regarding DTG distribution in the first trimester in early trophoblasts, extraembryonic membranes, coelomic or amniotic fluid, or in embryonic tissues when neurulation takes place.

Continued investigation into the variables and complex interactions that contribute to DTG-NTDs are needed to reassure women of childbearing age that risks associated with INSTI-based ART can be safely managed during pregnancy. Due to its cost effectiveness and favorable clinical profile, many low and middle-income countries have transitioned to DTG-based ART, but unanswered questions remain (Dorward, et al., 2018). According to a UNAIDS estimate, there were more than 38 million people living with HIV globally in 2021, and more than half (>20 million) of those infected were female (UNAIDS, 2022). In sub-Saharan Africa, young women of child-bearing age (15–24 years) were at highest risk, with >63% of all new HIV infections in 2021 occurring in this demographic, demonstrating the need for safe and effective ART during pregnancy. Despite the reduced safety signal for DTG-NTDs, pharmacovigilance and continued investigation in cell and animal-based models is needed to fully understand the factors that contribute to increased risk for DTG-NTDs. The same risk factors should also be considered for newer second-generation INSTI class antiretrovirals, including cabotegravir and bictegravir, especially as the use of long-acting injectable formulations become more widespread. A more comprehensive understanding of the underlying risk factors will better inform strategies for prevention.

Based on our findings, it would seem valuable going forward to measure plasma Mg^2+^ in women of childbearing age who are considering pregnancy and who are already on or considering starting an ART regimen with DTG or other INSTIs and then continue to monitor plasma Mg^2+^ longitudinally. Case-control NTD studies in women on DTG ART could include analysis of candidate genes involved in Mg^2+^ homeostasis. Other risk factors that impact plasma Mg^2+^ should also be evaluated in the context of women receiving INSTI-based ART, including diet, disease conditions, and medications. The use of prenatal vitamins that contain not only folate, but also meet the RDA for minerals in pregnant women, including magnesium, should be recommended. DTG is known to chelate metal cation-containing supplements (antacids) and other medications which decreases their absorption from the gut and reduces the DTG Cmax and AUC (Song et al., 2015; Enoki et al., 2021). Administration of DTG and prenatal vitamins containing Mg^2+^ would therefore need to be spaced accordingly to prevent this interaction. It has been proposed that dietary supplementation with divalent cations in patients living with HIV could lead to increased viral replication (Khan et al., 2020). Our studies were done in uninfected mice and did not account for the potential role of host-pathogen interactions as an additional variable in Mg^2+^ homeostasis. However, the interplay between maternal Mg^2+^ status, risk for DTG-NTDs and maintenance of adequate viral suppression could be tested in a humanized mouse model of HIV.

Collectively, key findings from our studies and others demonstrate a connection between early (pre-gastrulation) exposure to DTG and the potential for disruption of neural tube closure. In animal models, NTDs were not observed when DTG exposure started post-conception (Stanislaus et al., 2020; Posobiec et al., 2021), a timeframe that may not have been long enough to perturb Mg^2+^ homeostasis prior to neurulation. In addition, the animals were healthy and maintained on a nutritionally complete diet, meaning adverse effects may not have been detected even if DTG exposure had started earlier. Our findings highlight the multifactorial nature of NTDs and the importance of adequate Mg^2+^ during pregnancy. Overall, it seems likely that in healthy individuals on a Mg^2+^ sufficient diet, there is minimal risk for DTG-NTDs. However, many confounding factors exist in genetically diverse human populations with variable health and nutritional status that could contribute to subclinical hypomagnesemia and hidden risk. Any underlying factors that can be identified and modified to mitigate the chances of a woman on DTG-based ART having a child with a NTD warrant further investigation. It seems it might just be about finding the right balance.

## Data Availability

The original contributions presented in the study and data supporting the conclusions are included in the article and Supplementary Materials; further inquiries can be directed to the corresponding authors. The mouse whole exome sequencing data (BioProject ID: PRJNA978612) are available at: http://www.ncbi.nlm.nih.gov/bioproject/978612.

## References

[B1] Abraham M. B. Li D. Tang D. O'Connell S. M. McKenzie F. Lim E. M. (2017). Short stature and hypoparathyroidism in a child with Kenny-Caffey syndrome type 2 due to a novel mutation inFAM111A gene. Int. J. Pediatr. Endocrinol. 2017, 1. 10.1186/s13633-016-0041-7 28138333 PMC5264330

[B2] Adams J. B. Sorenson J. C. Pollard E. L. Kirby J. K. Audhya T. (2021). Evidence-based recommendations for an optimal prenatal supplement for women in the U.S., Part Two: Minerals. Nutrients 13 (6), 1849. 10.3390/nu13061849 34071548 PMC8229801

[B3] Alberts J. Rheeder J. Gelderblom W. Shephard G. Burger H. M. (2019). Rural subsistence maize farming in south Africa: Risk assessment and intervention models for reduction of exposure to fumonisin mycotoxins. Toxins 11 (6), 334. 10.3390/toxins11060334 31212811 PMC6628387

[B4] Allache R. Lachance S. Guyot M. C. De Marco P. Merello E. Justice M. J. (2014). Novel mutations in Lrp6 orthologs in mouse and human neural tube defects affect a highly dosage-sensitive Wnt non-canonical planar cell polarity pathway. Hum. Mol. Genet. 23 (7), 1687–1699. 10.1093/hmg/ddt558 24203697 PMC3943515

[B5] Allache R. Wang M. De Marco P. Merello E. Capra V. Kibar Z. (2015). Genetic studies of ANKRD6 as a molecular switch between Wnt signaling pathways in human neural tube defects. Birth defects Res. Part A, Clin. Mol. Teratol. 103 (1), 20–26. 10.1002/bdra.23273 25200652

[B6] Andersson E. R. Bryjova L. Biris K. Yamaguchi T. P. Arenas E. Bryja V. (2010). Genetic interaction between Lrp6 and Wnt5a during mouse development. Dev. Dyn. official Publ. Am. Assoc. Anatomists 239 (1), 237–245. 10.1002/dvdy.22101 PMC7282147 19795512

[B7] Andre P. Song H. Kim W. Kispert A. Yang Y. (2015). Wnt5a and Wnt11 regulate mammalian anterior-posterior axis elongation. Dev. Camb. Engl. 142 (8), 1516–1527. 10.1242/dev.119065 PMC4392599 25813538

[B8] Andrews P. A. McNerney M. E. DeGeorge J. J. (2019). Exposure assessments in reproductive and developmental toxicity testing: An IQ-DruSafe industry survey on current practices and experiences in support of exposure-based high dose selection. Regul. Toxicol. Pharmacol. RTP 107, 104413. 10.1016/j.yrtph.2019.104413 31229519

[B9] Atsmon J. Dolev E. (2005). Drug-induced hypomagnesaemia: Scope and management. Drug Saf. 28 (9), 763–788. 10.2165/00002018-200528090-00003 16119971

[B10] Atta C. A. Fiest K. M. Frolkis A. D. Jette N. Pringsheim T. St Germaine-Smith C. (2016). Global birth prevalence of spina bifida by folic acid fortification status: A systematic review and meta-analysis. Am. J. public health 106 (1), e24–e34. 10.2105/AJPH.2015.302902 26562127 PMC4695937

[B11] Bade A. N. McMillan J. M. Liu Y. Edagwa B. J. Gendelman H. E. (2021). Dolutegravir inhibition of matrix metalloproteinases affects mouse neurodevelopment. Mol. Neurobiol. 58 (11), 5703–5721. 10.1007/s12035-021-02508-5 34390469 PMC8599359

[B12] Bailin S. S. Koethe J. R. (2023). Diabetes in HIV: The link to weight gain. Curr. HIV/AIDS Rep. 20 (1), 9–18. 10.1007/s11904-022-00642-w 36418528 PMC10184162

[B13] Bardgett M. E. Schultheis P. J. McGill D. L. Richmond R. E. Wagge J. R. (2005). Magnesium deficiency impairs fear conditioning in mice. Brain Res. 1038 (1), 100–106. 10.1016/j.brainres.2005.01.020 15748878

[B14] Barreca M. L. Iraci N. De Luca L. Chimirri A. (2009). Induced-fit docking approach provides insight into the binding mode and mechanism of action of HIV-1 integrase inhibitors. ChemMedChem 4 (9), 1446–1456. 10.1002/cmdc.200900166 19544345

[B15] Barton C. H. Pahl M. Vaziri N. D. Cesario T. (1984). Renal magnesium wasting associated with amphotericin B therapy. Am. J. Med. 77 (3), 471–474. 10.1016/0002-9343(84)90106-2 6475987

[B16] Baumholtz A. I. De Marco P. Capra V. Ryan A. K. (2020). Functional validation of CLDN variants identified in a neural tube defect cohort demonstrates their contribution to neural tube defects. Front. Neurosci. 14, 664. 10.3389/fnins.2020.00664 32760237 PMC7372130

[B17] Beaudin A. E. Abarinov E. V. Noden D. M. Perry C. A. Chu S. Stabler S. P. (2011). Shmt1 and de novo thymidylate biosynthesis underlie folate-responsive neural tube defects in mice. Am. J. Clin. Nutr. 93 (4), 789–798. 10.3945/ajcn.110.002766 21346092 PMC3057548

[B18] Bennetto-Hood C. Tabolt G. Savina P. Acosta E. P. (2014). A sensitive HPLC-MS/MS method for the determination of dolutegravir in human plasma. J. Chromatogr. B, Anal. Technol. Biomed. life Sci. 945-946, 225–232. 10.1016/j.jchromb.2013.11.054 PMC4229012 24361860

[B19] Bollen P. Freriksen J. Konopnicki D. Weizsäcker K. Hidalgo Tenorio C. Moltó J. (2021). The effect of pregnancy on the pharmacokinetics of total and unbound dolutegravir and its main metabolite in women living with human immunodeficiency virus. Clin. Infect. Dis. 72 (1), 121–127. 10.1093/cid/ciaa006 32103260

[B20] Bosman W. Hoenderop J. G. J. de Baaij J. H. F. (2021). Genetic and drug-induced hypomagnesemia: Different cause, same mechanism. Proc. Nutr. Soc. 80 (3), 327–338. 10.1017/S0029665121000926 33906702

[B21] Bryja V. Andersson E. R. Schambony A. Esner M. Bryjová L. Biris K. K. (2009). The extracellular domain of Lrp5/6 inhibits noncanonical Wnt signaling *in vivo* . Mol. Biol. Cell 20 (3), 924–936. 10.1091/mbc.e08-07-0711 19056682 PMC2633404

[B22] Cabrera R. M. Souder J. P. Steele J. W. Yeo L. Tukeman G. Gorelick D. A. (2019). The antagonism of folate receptor by dolutegravir: Developmental toxicity reduction by supplemental folic acid. AIDS Lond. Engl. 33 (13), 1967–1976. 10.1097/QAD.0000000000002289 PMC6774845 31259764

[B23] Campbell J. Wathen N. Perry G. Soneji S. Sourial N. Chard T. (1993). The coelomic cavity: An important site of materno-fetal nutrient exchange in the first trimester of pregnancy. Br. J. obstetrics Gynaecol. 100 (8), 765–767. 10.1111/j.1471-0528.1993.tb14271.x 8399018

[B24] Chavez-Abiega S. Mos I. Centeno P. P. Elajnaf T. Schlattl W. Ward D. T. (2020). Sensing extracellular calcium - an insight into the structure and function of the calcium-sensing receptor (CaSR). Adv. Exp. Med. Biol. 1131, 1031–1063. 10.1007/978-3-030-12457-1_41 31646544

[B25] Chubanov V. Ferioli S. Wisnowsky A. Simmons D. G. Leitzinger C. Einer C. (2016). Epithelial magnesium transport by TRPM6 is essential for prenatal development and adult survival. eLife 5, e20914. 10.7554/eLife.20914 27991852 PMC5218537

[B26] Chubanov V. Gudermann T. Schlingmann K. P. (2005). Essential role for TRPM6 in epithelial magnesium transport and body magnesium homeostasis. Pflugers Archiv Eur. J. physiology 451 (1), 228–234. 10.1007/s00424-005-1470-y 16075242

[B27] Claverie-Martin F. Perdomo-Ramirez A. Garcia-Nieto V. (2021). Hereditary kidney diseases associated with hypomagnesemia. Kidney Res. Clin. Pract. 40 (4), 512–526. 10.23876/j.krcp.21.112 34784661 PMC8685365

[B28] Czeizel A. E. Dudás I. (1992). Prevention of the first occurrence of neural-tube defects by periconceptional vitamin supplementation. N. Engl. J. Med. 327 (26), 1832–1835. 10.1056/NEJM199212243272602 1307234

[B29] Dalton L. M. Ní Fhloinn D. M. Gaydadzhieva G. T. Mazurkiewicz O. M. Leeson H. Wright C. P. (2016). Magnesium in pregnancy. Nutr. Rev. 74 (9), 549–557. 10.1093/nutrit/nuw018 27445320

[B30] Darnton-Hill I. Mkparu U. C. (2015). Micronutrients in pregnancy in low- and middle-income countries. Nutrients 7 (3), 1744–1768. 10.3390/nu7031744 25763532 PMC4377879

[B31] de Baaij J. H. Hoenderop J. G. Bindels R. J. (2015). Magnesium in man: Implications for health and disease. Physiol. Rev. 95 (1), 1–46. 10.1152/physrev.00012.2014 25540137

[B32] De Clercq K. Pérez-García V. Van Bree R. Pollastro F. Peeraer K. Voets T. (2021). Mapping the expression of transient receptor potential channels across murine placental development. Cell. Mol. life Sci. CMLS 78 (11), 4993–5014. 10.1007/s00018-021-03837-3 33884443 PMC8233283

[B33] Dong Y. Wang X. Zhang J. Guan Z. Xu L. Wang J. (2015). Raltitrexed's effect on the development of neural tube defects in mice is associated with DNA damage, apoptosis, and proliferation. Mol. Cell. Biochem. 398 (1-2), 223–231. 10.1007/s11010-014-2222-0 25245820

[B34] Dorward J. Lessells R. Drain P. K. Naidoo K. de Oliveira T. Pillay Y. (2018). Dolutegravir for first-line antiretroviral therapy in low-income and middle-income countries: Uncertainties and opportunities for implementation and research. lancet. HIV 5 (7), e400–e404. 10.1016/S2352-3018(18)30093-6 29884404 PMC6063784

[B35] Downie M. L. Alexander R. T. (2022). Molecular mechanisms altering tubular calcium reabsorption. Pediatr. Nephrol. Berl. Ger. 37 (4), 707–718. 10.1007/s00467-021-05049-0 33796889

[B36] Dunlevy L. P. Chitty L. S. Burren K. A. Doudney K. Stojilkovic-Mikic T. Stanier P. (2007). Abnormal folate metabolism in foetuses affected by neural tube defects. Brain a J. neurology 130 (4), 1043–1049. 10.1093/brain/awm028 PMC7614420 17438019

[B37] Elin R. J. (1988). Magnesium metabolism in health and disease. Disease-a-month DM 34 (4), 161–218. 10.1016/0011-5029(88)90013-2 3282851

[B38] Enoki Y. Kishi N. Sakamoto K. Uchiyama E. Hayashi Y. Suzuki N. (2021). Multivalent cation and polycation polymer preparations influence pharmacokinetics of dolutegravir via chelation-type drug interactions. Drug metabolism Pharmacokinet. 37, 100371. 10.1016/j.dmpk.2020.11.006 33556698

[B39] European Medicines Agency ICH S5 (R3) (2020) Guidelines on reproductive toxicology: Detection of toxicity to reproduction for human pharmaceuticals. Available at: https://www.ema.europa.eu/en/documents/scientific-guideline/ich-s5-r3-guideline-reproductive-toxicology-detection-toxicity-reproduction-human-pharmaceuticals_en.pdf

[B40] Feng J. Wang H. Jing Z. Wang Y. Cheng Y. Wang W. (2020). Role of magnesium in type 2 diabetes mellitus. Biol. trace Elem. Res. 196 (1), 74–85. 10.1007/s12011-019-01922-0 31713111

[B41] Fine D. A. Rozenblatt-Rosen O. Padi M. Korkhin A. James R. L. Adelmant G. (2012). Identification of FAM111A as an SV40 host range restriction and adenovirus helper factor. PLoS Pathog. 8 (10), e1002949. 10.1371/journal.ppat.1002949 23093934 PMC3475652

[B42] Finnell R. H. Caiaffa C. D. Kim S. E. Lei Y. Steele J. Cao X. (2021). Gene environment interactions in the etiology of neural tube defects. Front. Genet. 12, 659612. 10.3389/fgene.2021.659612 34040637 PMC8143787

[B43] Gearhart M. O. Sorg T. B. (1993). Foscarnet-induced severe hypomagnesemia and other electrolyte disorders. Ann. Pharmacother. 27 (3), 285–289. 10.1177/106002809302700304 8384030

[B44] Gelineau-van Waes J. Rainey M. A. Maddox J. R. Voss K. A. Sachs A. J. Gardner N. M. (2012). Increased sphingoid base-1-phosphates and failure of neural tube closure after exposure to fumonisin or FTY720. Part A, Clin. Mol. Teratol. 94 (10), 790–803. 10.1002/bdra.23074 22991331

[B45] Gelineau-van Waes J. Starr L. Maddox J. Aleman F. Voss K. A. Wilberding J. (2005). Maternal fumonisin exposure and risk for neural tube defects: Mechanisms in an *in vivo* mouse model. Birth defects Res. Part A, Clin. Mol. Teratol. 73 (7), 487–497. 10.1002/bdra.20148 15959874

[B46] Gibson R. S. Raboy V. King J. C. (2018). Implications of phytate in plant-based foods for iron and zinc bioavailability, setting dietary requirements, and formulating programs and policies. Nutr. Rev. 76 (11), 793–804. 10.1093/nutrit/nuy028 30010865

[B47] Gilmore J. C. Hoque M. T. Dai W. Mohan H. Dunk C. Serghides L. (2022). Interaction between dolutegravir and folate transporters and receptor in human and rodent placenta. EBioMedicine 75, 103771. 10.1016/j.ebiom.2021.103771 34954655 PMC8715299

[B48] Groenen P. M. van Rooij I. A. Peer P. G. Ocké M. C. Zielhuis G. A. Steegers-Theunissen R. P. (2004). Low maternal dietary intakes of iron, magnesium, and niacin are associated with spina bifida in the offspring. J. Nutr. 134 (6), 1516–1522. 10.1093/jn/134.6.1516 15173422

[B49] Groenestege W. M. Hoenderop J. G. van den Heuvel L. Knoers N. Bindels R. J. (2006). The epithelial Mg2+ channel transient receptor potential melastatin 6 is regulated by dietary Mg2+ content and estrogens. J. Am. Soc. Nephrol. JASN 17 (4), 1035–1043. 10.1681/ASN.2005070700 16524949

[B50] Günzel D. Yu A. S. (2009). Function and regulation of claudins in the thick ascending limb of Henle. Pflugers Archiv Eur. J. physiology 458 (1), 77–88. 10.1007/s00424-008-0589-z 18795318 PMC2666100

[B51] Gupta R. K. Gangoliya S. S. Singh N. K. (2015). Reduction of phytic acid and enhancement of bioavailable micronutrients in food grains. J. food Sci. Technol. 52 (2), 676–684. 10.1007/s13197-013-0978-y 25694676 PMC4325021

[B52] Harris M. J. Juriloff D. M. Biddle F. G. (1984). Cortisone cure of the lidgap defect in fetal mice: A dose-response and time-response study. Teratology 29 (2), 287–295. 10.1002/tera.1420290215 6740513

[B53] Hoffmann S. Pentakota S. Mund A. Haahr P. Coscia F. Gallo M. (2020). FAM111 protease activity undermines cellular fitness and is amplified by gain-of-function mutations in human disease. EMBO Rep. 21 (10), e50662. 10.15252/embr.202050662 32776417 PMC7534640

[B54] Huycke M. M. Naguib M. T. Stroemmel M. M. Blick K. Monti K. Martin-Munley S. (2000). A double-blind placebo-controlled crossover trial of intravenous magnesium sulfate for foscarnet-induced ionized hypocalcemia and hypomagnesemia in patients with AIDS and cytomegalovirus infection. Antimicrob. agents Chemother. 44 (8), 2143–2148. 10.1128/AAC.44.8.2143-2148.2000 10898688 PMC90026

[B55] Ilenwabor B. P. Schigt H. Kompatscher A. Bos C. Zuidscherwoude M. van der Eerden B. C. J. (2022). FAM111A is dispensable for electrolyte homeostasis in mice. Sci. Rep. 12 (1), 10211. 10.1038/s41598-022-14054-8 35715480 PMC9205974

[B56] Isaković J. Šimunić I. Jagečić D. Hribljan V. Mitrečić D. (2022). Overview of neural tube defects: Gene-environment interactions, preventative approaches and future perspectives. Biomedicines 10 (5), 965. 10.3390/biomedicines10050965 35625701 PMC9138472

[B57] Isojima T. Doi K. Mitsui J. Oda Y. Tokuhiro E. Yasoda A. (2014). A recurrent de novo FAM111A mutation causes Kenny-Caffey syndrome type 2. J. bone mineral Res. 29 (4), 992–998. 10.1002/jbmr.2091 23996431

[B58] Jacobson M. A. Gambertoglio J. G. Aweeka F. T. Causey D. M. Portale A. A. (1991). Foscarnet-induced hypocalcemia and effects of foscarnet on calcium metabolism. J. Clin. Endocrinol. metabolism 72 (5), 1130–1135. 10.1210/jcem-72-5-1130 1827127

[B59] Jahnen-Dechent W. Ketteler M. (2012). Magnesium basics. Clin. kidney J. 5 (1), i3–i14. 10.1093/ndtplus/sfr163 26069819 PMC4455825

[B60] Jin J. Desai B. N. Navarro B. Donovan A. Andrews N. C. Clapham D. E. (2008). Deletion of Trpm7 disrupts embryonic development and thymopoiesis without altering Mg2+ homeostasis. Sci. (New York, N.Y.) 322 (5902), 756–760. 10.1126/science.1163493 PMC2605283 18974357

[B61] Katopodis P. Karteris E. Katopodis K. P. (2020). Pathophysiology of drug-induced hypomagnesaemia. Drug Saf. 43 (9), 867–880. 10.1007/s40264-020-00947-y 32399868

[B62] Kawasuji T. Fuji M. Yoshinaga T. Sato A. Fujiwara T. Kiyama R. (2006). A platform for designing HIV integrase inhibitors. Part 2: A two-metal binding model as a potential mechanism of HIV integrase inhibitors. Bioorg. Med. Chem. 14 (24), 8420–8429. 10.1016/j.bmc.2006.08.043 17005407

[B63] Khan N. Chen X. Geiger J. D. (2020). Role of divalent cations in HIV-1 replication and pathogenicity. Viruses 12 (4), 471. 10.3390/v12040471 32326317 PMC7232465

[B64] Kirkwood-Johnson L. Katayama N. Marikawa Y. (2021). Dolutegravir impairs stem cell-based 3D morphogenesis models in a manner dependent on dose and timing of exposure: An implication for its developmental toxicity. Toxicol. Sci. 184 (2), 191–203. 10.1093/toxsci/kfab112 34515794 PMC8783619

[B65] Kojima Y. Machida Y. Palani S. Caulfield T. R. Radisky E. S. Kaufmann S. H. (2020). FAM111A protects replication forks from protein obstacles via its trypsin-like domain. Nat. Commun. 11 (1), 1318. 10.1038/s41467-020-15170-7 32165630 PMC7067828

[B66] Komiya Y. Bai Z. Cai N. Lou L. Al-Saadi N. Mezzacappa C. (2017). A nonredundant role for the TRPM6 channel in neural tube closure. Sci. Rep. 7 (1), 15623. 10.1038/s41598-017-15855-y 29142255 PMC5688082

[B67] Komiya Y. Runnels L. W. (2015). TRPM channels and magnesium in early embryonic development. Int. J. Dev. Biol. 59 (7-9), 281–288. 10.1387/ijdb.150196lr 26679946 PMC4685952

[B68] Komiya Y. Su L. T. Chen H. C. Habas R. Runnels L. W. (2014). Magnesium and embryonic development. Magnesium Res. 27 (1), 1–8. 10.1684/mrh.2014.0356 PMC4207262 24721994

[B69] Koren G. Pastuszak A. Ito S. (1998). Drugs in pregnancy. N. Engl. J. Med. 338 (16), 1128–1137. 10.1056/NEJM199804163381607 9545362

[B70] Lamorde M. Atwiine M. Owarwo N. C. Ddungu A. Laker E. O. Mubiru F. (2020). Dolutegravir-associated hyperglycaemia in patients with HIV. lancet HIV 7 (7), e461–e462. 10.1016/S2352-3018(20)30042-4 32105626

[B71] Liu G. Hu X. Chakrabarty S. (2010). Vitamin D mediates its action in human colon carcinoma cells in a calcium-sensing receptor-dependent manner: Downregulates malignant cell behavior and the expression of thymidylate synthase and survivin and promotes cellular sensitivity to 5-FU. Int. J. cancer 126 (3), 631–639. 10.1002/ijc.24762 19621386

[B72] Liu G. Hu X. Premkumar L. Chakrabarty S. (2011). Nifedipine synergizes with calcium in activating the calcium sensing receptor, suppressing the expression of thymidylate synthase and survivin and promoting sensitivity to fluorouracil in human colon carcinoma cells. Mol. Carcinog. 50 (12), 922–930. 10.1002/mc.20752 21374737

[B73] Liu W. Su L. T. Khadka D. K. Mezzacappa C. Komiya Y. Sato A. (2011). TRPM7 regulates gastrulation during vertebrate embryogenesis. Dev. Biol. 350 (2), 348–357. 10.1016/j.ydbio.2010.11.034 21145885 PMC3292586

[B74] Lupo P. J. Canfield M. A. Chapa C. Lu W. Agopian A. J. Mitchell L. E. (2012). Diabetes and obesity-related genes and the risk of neural tube defects in the national birth defects prevention study. Am. J. Epidemiol. 176 (12), 1101–1109. 10.1093/aje/kws190 23132673 PMC3571234

[B75] MacLeod R. J. Hayes M. Pacheco I. (2007). Wnt5a secretion stimulated by the extracellular calcium-sensing receptor inhibits defective Wnt signaling in colon cancer cells. Am. J. physiology Gastrointest. liver physiology 293 (1), G403–G411. 10.1152/ajpgi.00119.2007 17463182

[B76] Mandelbrot L. Ceccaldi P. F. Duro D. Lê M. Pencolé L. Peytavin G. (2019). Placental transfer and tissue accumulation of dolutegravir in the *ex vivo* human cotyledon perfusion model. PloS one 14 (8), e0220323. 10.1371/journal.pone.0220323 31408460 PMC6692001

[B77] Marikawa Y. Chen H. R. Menor M. Deng Y. Alarcon V. B. (2020). Exposure-based assessment of chemical teratogenicity using morphogenetic aggregates of human embryonic stem cells. Reprod. Toxicol. (Elmsford, N.Y.) 91, 74–91. 10.1016/j.reprotox.2019.10.004 PMC6980740 31711903

[B78] Marolt G. Gričar E. Pihlar B. Kolar M. (2020). Complex Formation of phytic acid with selected monovalent and divalent metals. Front. Chem. 8, 582746. 10.3389/fchem.2020.582746 33173770 PMC7539747

[B79] Mathew A. A. Panonnummal R. (2021). Magnesium'-the master cation-as a drug-possibilities and evidences. Biometals 34 (5), 955–986. 10.1007/s10534-021-00328-7 34213669 PMC8249833

[B80] McCann K. Shah S. Hindley L. Hill A. Qavi A. Simmons B. (2021). Implications of weight gain with newer anti-retrovirals: 10-year predictions of cardiovascular disease and diabetes. AIDS Lond. Engl. 35 (10), 1657–1665. 10.1097/QAD.0000000000002930 33927086

[B81] Mimouni F. Miodovnik M. Tsang R. C. Callahan J. Shaul P. (1987). Decreased amniotic fluid magnesium concentration in diabetic pregnancy. Obstetrics Gynecol. 69 (1), 12–14. 3796911

[B82] Min S. Song I. Borland J. Chen S. Lou Y. Fujiwara T. (2010). Pharmacokinetics and safety of S/GSK1349572, a next-generation HIV integrase inhibitor, in healthy volunteers. Antimicrob. agents Chemother. 54 (1), 254–258. 10.1128/AAC.00842-09 19884365 PMC2798521

[B83] Mohan H. Lenis M. G. Laurette E. Y. Tejada O. Sanghvi T. Leung K. Y. (2021). Dolutegravir in pregnant mice is associated with increased rates of fetal defects at therapeutic but not at supratherapeutic levels. EBioMedicine 63, 103167. 10.1016/j.ebiom.2020.103167 33341441 PMC7753150

[B84] Mos I. Jacobsen S. E. Foster S. R. Bräuner-Osborne H. (2019). Calcium-Sensing receptor internalization isβ-arrestin-dependent and modulated by allosteric ligands. Mol. Pharmacol. 96 (4), 463–474. 10.1124/mol.119.116772 31399503

[B85] Moss L. Wagner D. Kanaoka E. Olson K. Yueh Y. L. Bowers G. D. (2015). The comparative disposition and metabolism of dolutegravir, a potent HIV-1 integrase inhibitor, in mice, rats, and monkeys. Xenobiotica; fate foreign Compd. Biol. Syst. 45 (1), 60–70. 10.3109/00498254.2014.942409 25034010

[B86] MRC Vitamin Study Research Group (1991). Prevention of neural tube defects: Results of the medical research Council vitamin study. Lancet (London, Engl. 338 (8760), 131–137. 1677062

[B87] Müller R. Steffensen T. Krstić N. Cain M. A. (2021). Report of a novel variant in the FAM111A gene in a fetus with multiple anomalies including gracile bones, hypoplastic spleen, and hypomineralized skull. Am. J. Med. Genet. Part A 185 (6), 1903–1907. 10.1002/ajmg.a.62182 33750016

[B88] Mulligan N. Best B. M. Wang J. Capparelli E. V. Stek A. Barr E. IMPAACT P1026s Protocol Team (2018). Dolutegravir pharmacokinetics in pregnant and postpartum women living with HIV. AIDS Lond. Engl. 32 (6), 729–737. 10.1097/QAD.0000000000001755 PMC5854536 29369162

[B89] Nie M. Oravcová M. Jami-Alahmadi Y. Wohlschlegel J. A. Lazzerini-Denchi E. Boddy M. N. (2021). FAM111A induces nuclear dysfunction in disease and viral restriction. EMBO Rep. 22 (2), e50803. 10.15252/embr.202050803 33369867 PMC7857424

[B90] Omotayo O. P. Omotayo A. O. Mwanza M. Babalola O. O. (2019). Prevalence of mycotoxins and their consequences on human health. Toxicol. Res. 35 (1), 1–7. 10.5487/TR.2019.35.1.001 30766652 PMC6354945

[B91] Osei-Sarfo K. Gudas L. J. (2014). Retinoic acid suppresses the canonical Wnt signaling pathway in embryonic stem cells and activates the noncanonical Wnt signaling pathway. Stem cells Dayt. Ohio) 32 (8), 2061–2071. 10.1002/stem.1706 PMC4106995 24648413

[B92] Panda D. Fernandez D. J. Lal M. Buehler E. Moss B. (2017). Triad of human cellular proteins, IRF2, FAM111A, and RFC3, restrict replication of orthopoxvirus SPI-1 host-range mutants. Proc. Natl. Acad. Sci. U. S. A. 114 (14), 3720–3725. 10.1073/pnas.1700678114 28320935 PMC5389286

[B93] Pelczyńska M. Moszak M. Bogdański P. (2022). The role of magnesium in the pathogenesis of metabolic disorders. Nutrients 14 (9), 1714. 10.3390/nu14091714 35565682 PMC9103223

[B94] Pi M. Oakley R. H. Gesty-Palmer D. Cruickshank R. D. Spurney R. F. Luttrell L. M. (2005). Beta-arrestin- and G protein receptor kinase-mediated calcium-sensing receptor desensitization. Mol. Endocrinol. Baltim. Md 19 (4), 1078–1087. 10.1210/me.2004-0450 15637145

[B95] Posobiec L. M. Chapman S. P. Murzyn S. F. Rendemonti J. E. Stanislaus D. J. Romach E. H. (2021). No developmental toxicity observed with dolutegravir in rat whole embryo culture. Birth defects Res. 113 (16), 1190–1197. 10.1002/bdr2.1949 34453500

[B96] Qian D. Jones C. Rzadzinska A. Mark S. Zhang X. Steel K. P. (2007). Wnt5a functions in planar cell polarity regulation in mice. Dev. Biol. 306 (1), 121–133. 10.1016/j.ydbio.2007.03.011 17433286 PMC1978180

[B97] Qin X. Yue Z. Sun B. Yang W. Xie J. Ni E. (2013). Sphingosine and FTY720 are potent inhibitors of the transient receptor potential melastatin 7 (TRPM7) channels. Br. J. Pharmacol. 168 (6), 1294–1312. 10.1111/bph.12012 23145923 PMC3596637

[B98] Ren K. Zhu Y. Sun H. Li S. Duan X. Li S. (2021). IRF2 inhibits ZIKV replication by promoting FAM111A expression to enhance the host restriction effect of RFC3. Virology J. 18 (1), 256. 10.1186/s12985-021-01724-8 34930359 PMC8691090

[B99] Riley R. T. Showker J. L. Lee C. M. Zipperer C. E. Mitchell T. R. Voss K. A. (2015a). A blood spot method for detecting fumonisin-induced changes in putative sphingolipid biomarkers in LM/Bc mice and humans. Food Addit. Contam. Part A, Chem. analysis, control, Expo. risk Assess. 32 (6), 934–949. 10.1080/19440049.2015.1027746 25833119

[B100] Riley R. T. Torres O. Matute J. Gregory S. G. Ashley-Koch A. E. Showker J. L. (2015b). Evidence for fumonisin inhibition of ceramide synthase in humans consuming maize-based foods and living in high exposure communities in Guatemala. Mol. Nutr. food Res. 59 (11), 2209–2224. 10.1002/mnfr.201500499 26264677 PMC4956729

[B101] Rodelo-Haad C. Pendón-Ruiz de Mier M. V. Díaz-Tocados J. M. Martin-Malo A. Santamaria R. Muñoz-Castañeda J. R. (2020). The role of disturbed Mg homeostasis in chronic kidney disease comorbidities. Front. Cell Dev. Biol. 8, 543099. 10.3389/fcell.2020.543099 33282857 PMC7688914

[B102] Rosanoff A. West C. Elin R. J. Micke O. Baniasadi S. Barbagallo M. (2022). Recommendation on an updated standardization of serum magnesium reference ranges.Eur. J. Nutr., 61(7), 3697–3706. 10.1007/s00394-022-02916-w 35689124 PMC9186275

[B103] Rosato S. Unger S. Campos-Xavier B. Caraffi S. G. Beltrami L. Pollazzon M. (2022). Clinical and molecular diagnosis of osteocraniostenosis in fetuses and newborns: Prenatal ultrasound, clinical, radiological and pathological features. Genes 13 (2), 261. 10.3390/genes13020261 35205306 PMC8871755

[B104] Runnels L. W. Komiya Y. (2020). TRPM6 and TRPM7: Novel players in cell intercalation during vertebrate embryonic development. Dev. Dyn. 249 (8), 912–923. 10.1002/dvdy.182 32315468

[B105] Sadler T. W. Merrill A. H. Stevens V. L. Sullards M. C. Wang E. Wang P. (2002). Prevention of fumonisin B1-induced neural tube defects by folic acid. Teratology 66 (4), 169–176. 10.1002/tera.10089 12353213

[B106] Saraç M. Önalan E. Bakal Ü. Tartar T. Aydın M. Orman A. (2016). Magnesium-permeableTRPM6 polymorphisms in patients with meningomyelocele. SpringerPlus 5 (1), 1703. 10.1186/s40064-016-3395-7 27757375 PMC5047867

[B107] Schalkwijk S. Greupink R. Colbers A. P. Wouterse A. C. Verweij V. G. van Drongelen J. (2016). Placental transfer of the HIV integrase inhibitor dolutegravir in an *ex vivo* human cotyledon perfusion model. J. Antimicrob. Chemother. 71 (2), 480–483. 10.1093/jac/dkv358 26538508

[B108] Schlegel R. N. Cuffe J. S. Moritz K. M. Paravicini T. M. (2015). Maternal hypomagnesemia causes placental abnormalities and fetal and postnatal mortality. Placenta 36 (7), 750–758. 10.1016/j.placenta.2015.03.011 25924939

[B109] Schlingmann K. P. Gudermann T. (2005). A critical role of TRPM channel-kinase for human magnesium transport. J. physiology 566 (2), 301–308. 10.1113/jphysiol.2004.080200 PMC1464747 15845589

[B110] Schlingmann K. P. Waldegger S. Konrad M. Chubanov V. Gudermann T. (2007). TRPM6 and TRPM7--Gatekeepers of human magnesium metabolism. Biochimica biophysica acta 1772 (8), 813–821. 10.1016/j.bbadis.2007.03.009 17481860

[B111] Schlingmann K. P. Weber S. Peters M. Niemann Nejsum L. Vitzthum H. Klingel K. (2002). Hypomagnesemia with secondary hypocalcemia is caused by mutations in TRPM6, a new member of the TRPM gene family. Nat. Genet. 31 (2), 166–170. 10.1038/ng889 12032568

[B112] Schuchardt J. P. Hahn A. (2017). Intestinal absorption and factors influencing bioavailability of magnesium-an update. Curr. Nutr. food Sci. 13 (4), 260–278. 10.2174/1573401313666170427162740 29123461 PMC5652077

[B113] Shah B. M. Schafer J. J. Desimone J. A. Jr (2014). Dolutegravir: A new integrase strand transfer inhibitor for the treatment of HIV. Pharmacotherapy 34 (5), 506–520. 10.1002/phar.1386 24347095

[B114] Shariatmadari M. Peyronnet J. Papachristou P. Horn Z. Sousa K. M. Arenas E. (2005). Increased Wnt levels in the neural tube impair the function of adherens junctions during neurulation. Mol. Cell. Neurosci. 30 (3), 437–451. 10.1016/j.mcn.2005.08.008 16154760

[B115] Shaw G. M. Todoroff K. Schaffer D. M. Selvin S. (1999). Periconceptional nutrient intake and risk for neural tube defect-affected pregnancies. Epidemiol. Camb. Mass.) 10 (6), 711–716. 10.1097/00001648-199911000-00011 10535785

[B116] Shi D. L. (2022). Wnt/planar cell polarity signaling controls morphogenetic movements of gastrulation and neural tube closure. Cell. Mol. life Sci. CMLS 79 (12), 586. 10.1007/s00018-022-04620-8 36369349 PMC11803072

[B117] Smith M. R. Mohan H. Ajaykumar A. Hsieh A. Y. Y. Martineau L. Patel R. (2022). Second-generation human immunodeficiency virus integrase inhibitors induce differentiation dysregulation and exert toxic effects in human embryonic stem cell and mouse models. J. Infect. Dis. 226 (11), 1992–2001. 10.1093/infdis/jiac386 36124861 PMC10205620

[B118] Song I. Borland J. Arya N. Wynne B. Piscitelli S. (2015). Pharmacokinetics of dolutegravir when administered with mineral supplements in healthy adult subjects. J. Clin. Pharmacol. 55 (5), 490–496. 10.1002/jcph.439 25449994 PMC4407950

[B119] Stanislaus D. J. Posobiec L. M. Laffan S. B. Solomon H. M. Ziejewski M. K. Romach E. H. (2020). Absence of developmental and reproductive toxicity in animals exposed to dolutegravir. Birth defects Res. 112 (3), 245–261. 10.1002/bdr2.1635 31859466

[B120] Sun M. He Z. Jaisi D. P. (2021). Role of metal complexation on the solubility and enzymatic hydrolysis of phytate. PloS one 16 (8), e0255787. 10.1371/journal.pone.0255787 34388208 PMC8362945

[B121] Tan R. S. G. Lee C. H. L. Dimke H. Todd Alexander R. (2021). The role of calcium-sensing receptor signaling in regulating transepithelial calcium transport. Exp. Biol. Med. (Maywood, N.J.) 246 (22), 2407–2419. 10.1177/15353702211010415 PMC8606958 33926258

[B122] Tarnita R. M. Wilkie A. R. DeCaprio J. A. (2018). Contribution of DNA replication to the FAM111A-mediated simian virus 40 host range phenotype. J. virology 93 (1), e01330–18. 10.1128/JVI.01330-18 30333173 PMC6288344

[B123] Thuppal S. V. Jun S. Cowan A. Bailey R. L. (2017). The nutritional status of HIV-infected US adults. Curr. Dev. Nutr. 1 (10), e001636. 10.3945/cdn.117.001636 29955683 PMC5998784

[B124] Topol L. Jiang X. Choi H. Garrett-Beal L. Carolan P. J. Yang Y. (2003). Wnt-5a inhibits the canonical Wnt pathway by promoting GSK-3-independent beta-catenin degradation. J. Cell Biol. 162 (5), 899–908. 10.1083/jcb.200303158 12952940 PMC2172823

[B125] Toriello H. V. (2011). Policy statement on folic acid and neural tube defects. Genet. Med. 13 (6), 593–596. 10.1097/GIM.0b013e31821d4188 21552133

[B126] UNAIDS (2022) Global HIV and AIDS statistics – fact sheet. Available at: https://www.unaids.org/en/resources/fact-sheet

[B127] Unger S. Górna M. W. Le Béchec A. Do Vale-Pereira S. Bedeschi M. F. Geiberger S. (2013). FAM111A mutations result in hypoparathyroidism and impaired skeletal development. Am. J. Hum. Genet. 92 (6), 990–995. 10.1016/j.ajhg.2013.04.020 23684011 PMC3675238

[B128] Vashishth A. Ram S. Beniwal V. (2017). Cereal phytases and their importance in improvement of micronutrients bioavailability. 3 Biotech. 7 (1), 42. 10.1007/s13205-017-0698-5 PMC5428090 28444586

[B129] Viering D. H. H. M. de Baaij J. H. F. Walsh S. B. Kleta R. Bockenhauer D. (2017). Genetic causes of hypomagnesemia, a clinical overview. Pediatr. Nephrol. Berl. Ger. 32 (7), 1123–1135. 10.1007/s00467-016-3416-3 PMC5440500 27234911

[B130] Voets T. Nilius B. Hoefs S. van der Kemp A. W. Droogmans G. Bindels R. J. (2004). TRPM6 forms the Mg2+ influx channel involved in intestinal and renal Mg2+ absorption. J. Biol. Chem. 279 (1), 19–25. 10.1074/jbc.M311201200 14576148

[B131] Volcik K. A. Shaw G. M. Zhu H. Lammer E. J. Laurent C. Finnell R. H. (2003). Associations between polymorphisms within the thymidylate synthase gene and spina bifida. Birth defects Res. Part A, Clin. Mol. Teratol. 67 (11), 924–928. 10.1002/bdra.10029 14745930

[B133] Waitt C. Orrell C. Walimbwa S. Singh Y. Kintu K. Simmons B. (2019). Safety and pharmacokinetics of dolutegravir in pregnant mothers with HIV infection and their neonates: A randomised trial (DolPHIN-1 study). PLoS Med. 16 (9), e1002895. 10.1371/journal.pmed.1002895 31539371 PMC6754125

[B134] Walder R. Y. Landau D. Meyer P. Shalev H. Tsolia M. Borochowitz Z. (2002). Mutation of TRPM6 causes familial hypomagnesemia with secondary hypocalcemia. Nat. Genet. 31 (2), 171–174. 10.1038/ng901 12032570

[B135] Walder R. Y. Yang B. Stokes J. B. Kirby P. A. Cao X. Shi P. (2009). Mice defective in Trpm6 show embryonic mortality and neural tube defects. Hum. Mol. Genet. 18 (22), 4367–4375. 10.1093/hmg/ddp392 19692351 PMC2766295

[B136] Wang X. Cerrone M. Ferretti F. Castrillo N. Maartens G. McClure M. (2019). Pharmacokinetics of dolutegravir 100 mg once daily with rifampicin. Int. J. Antimicrob. agents 54 (2), 202–206. 10.1016/j.ijantimicag.2019.04.009 31002950

[B137] Wang X. Guan Z. Dong Y. Zhu Z. Wang J. Niu B. (2018). Inhibition of thymidylate synthase affects neural tube development in mice. Reprod. Toxicol. (Elmsford, N.Y.) 76, 17–25. 10.1016/j.reprotox.2017.12.007 29258758

[B138] Warkus E. L. L. Marikawa Y. (2017). Exposure-based validation of an *in vitro* gastrulation model for developmental toxicity assays. Toxicol. Sci. official J. Soc. Toxicol. 157 (1), 235–245. 10.1093/toxsci/kfx034 28184906

[B139] Wathen N. C. Delves H. T. Campbell D. J. Chard T. (1995). The coelomic cavity--a reservoir for metals. Am. J. obstetrics Gynecol. 173 (6), 1884–1888. 10.1016/0002-9378(95)90446-8 8610781

[B140] Wearne N. Davidson B. Blockman M. Swart A. Jones E. S. (2020). HIV, drugs and the kidney. Drugs context 9, 1–17. 10.7573/dic.2019-11-1 PMC7104683 32256631

[B141] Wells I. C. (2008). Evidence that the etiology of the syndrome containing type 2 diabetes mellitus results from abnormal magnesium metabolism. Can. J. physiology Pharmacol. 86 (1-2), 16–24. 10.1139/y07-122 18418443

[B142] Welter A. L. Machida Y. J. (2022). Functions and evolution of FAM111 serine proteases. Front. Mol. Biosci. 9, 1081166. 10.3389/fmolb.2022.1081166 36589246 PMC9798293

[B143] WHO (2019) World Health Organization revised guidelines for dolutegravir. Available at: https://www.who.int/news/item/22-07-2019-who-recommends-dolutegravir-as-preferred-hiv-treatment-option-in-all-populations

[B145] Woudenberg-Vrenken T. E. Sukinta A. van der Kemp A. W. Bindels R. J. Hoenderop J. G. (2011). Transient receptor potential melastatin 6 knockout mice are lethal whereas heterozygous deletion results in mild hypomagnesemia. Nephron. Physiol. 117 (2), p11–p19. 10.1159/000320580 20814221

[B146] Yamaguchi T. P. Bradley A. McMahon A. P. Jones S. (1999). A Wnt5a pathway underlies outgrowth of multiple structures in the vertebrate embryo. Dev. Camb. Engl. 126 (6), 1211–1223. 10.1242/dev.126.6.1211 10021340

[B147] Yli-Mattila T. Sundheim L. (2022). Fumonisins in african countries. Toxins 14 (6), 419. 10.3390/toxins14060419 35737080 PMC9228379

[B148] Yuan R. Korstanje R. Tsaih S. W. Schultz D. Godfrey D. (2008). Aging study: Blood chemistry for 32 inbred strains of mice. Maine: Mouse Phenome Database at The Jackson Laboratory.

[B149] Zamek-Gliszczynski M. J. Zhang X. Mudunuru J. Du Y. Chen J. L. Taskar K. S. (2019). Clinical extrapolation of the effects of dolutegravir and other HIV integrase inhibitors on folate transport pathways. Drug metabolism Dispos. Biol. fate Chem. 47 (8), 890–898. 10.1124/dmd.119.087635 31167838

[B150] Zash R. Holmes L. B. Diseko M. Jacobson D. Mayondi G. Mabuta J. (2022). Update on neural tube defects with antiretroviral exposure in the Tsepamo Study, Botswana. Presented at AIDS 2022 Montreal

[B151] Zash R. Holmes L. Diseko M. Jacobson D. L. Brummel S. Mayondi G. (2019). Neural-tube defects and antiretroviral treatment regimens in Botswana. N. Engl. J. Med. 381 (9), 827–840. 10.1056/NEJMoa1905230 31329379 PMC6995896

[B152] Zash R. Makhema J. Shapiro R. L. (2018). Neural-tube defects with dolutegravir treatment from the time of conception. N. Engl. J. Med. 379 (10), 979–981. 10.1056/NEJMc1807653 30037297 PMC6550482

[B153] Zhang Y. Huo M. Zhou J. Xie S. (2010). PKSolver: An add-in program for pharmacokinetic and pharmacodynamic data analysis in Microsoft Excel. Comput. methods programs Biomed. 99 (3), 306–314. 10.1016/j.cmpb.2010.01.007 20176408

[B154] Zohn I. E. Sarkar A. A. (2010). The visceral yolk sac endoderm provides for absorption of nutrients to the embryo during neurulation. Birth defects Res. Part A, Clin. Mol. Teratol. 88 (8), 593–600. 10.1002/bdra.20705 20672346

