# Supplementary material for: Gene-nutrient interactions that impact magnesium homeostasis increase risk for neural tube defects in mice exposed to dolutegravir
Source: Front Cell Dev Biol. 2023 Jun 12;11:1175917. doi: 10.3389/fcell.2023.1175917 (PMC10292217; doi:10.3389/fcell.2023.1175917)
Supplement: Supplementary file 1 [file DataSheet1.zip › Supplementary Material/Supplementary Data Sheet 1.docx]

Supplementary Material

Gene-Nutrient Interactions that Impact Magnesium Homeostasis Increase Risk for Neural Tube Defects in Mice Exposed to Dolutegravir

Gelineau-van Waes, J., van Waes, MA., Hallgren, J., Hulen, J., Bredehoeft, M., Ashley-Koch, AE., Krupp, D., Gregory, SG., Stessman, HA.

*** Correspondence:** Janée Gelineau-van Waes, [janeegelineau-vanwaes@creighton.edu](mailto:janeegelineau-vanwaes@creighton.edu)

# Table S1: Diet Comparison.

|  | **ENVIGO**  **Teklad Rodent Soy Diet 8904** | **NRC Requirement**  *Natural-ingredient, open-formula diet | **Mg^2+^ Deficient**  **TD.93106**  **Casein Diet** | **Mg^2+^ Control**  **TD.98341**  **Casein DIet** | **NRC Requirement**  *Purified ingredient diet |
| --- | --- | --- | --- | --- | --- |
| **MINERALS** | **%** | **%** | **%** | **%** | **%** |
| Magnesium | 0.3 | 0.18 | <0.003 | 0.05 | 0.05 |
| Calcium | 1.4 | 1.23 | 0.6 | 0.6 | 0.52 |
| Potassium | 1.0 | 0.85 | 0.95 | 0.95 | 0.36 |
|  | **mg/kg** | **mg/kg** | **mg/kg** | **mg/kg** | **mg/kg** |
| Copper | 25 | 16 | 12 | 12 | 6 |
| Iron | 300 | 255 | 250 | 250 | 35 |
| Manganese | 100 | 104 | 120 | 120 | 54 |
| Zinc | 80 | 50 | 56 | 56 | 30 |
| **VITAMINS** | **mg/kg** | **mg/kg** | **mg/kg** | **mg/kg** | **mg/kg** |
| Folic Acid | 3 | 4 | 2 | 2 | 2 |
| Biotin | 0.38 | 0.2 | 0.44 | 0.44 | 0.2 |
| Niacin | 63 | 82 | 99 | 99 | 30 |
| Pantothenic Acid | 21 | 21 | 66 | 66 | 16 |
| Riboflavin (B_2_) | 8 | 8 | 22 | 22 | 6 |
| Thiamin (B_1_) | 27 | 17 | 22 | 22 | 6 |
| Vit. B_6_ | 13 | 10 | 22 | 22 | 7 |
| Vit. B_12_ | .05 | .03 | .3 | .3 | .01 |
| Vit. K | 40 | 3 | 50 | 50 | .05 |
|  | **IU/kg** | **IU/kg** | **IU/kg** | **IU/kg** | **IU/kg** |
| Vit. A | 12,600 | 15,000 | 12,100 | 12,100 | 4000 |
| Vit. D_3_ | 2400 | 5000 | 2200 | 2200 | 1000 |
| Vit. E | 120 | 37 | 120 | 120 | 50 |

# Table S2: Variant Details by Mouse Strain.

|  | **SWV** | | **LM/Bc** | |
| --- | --- | --- | --- | --- |
| **Category** | **InDels** | **SNVs** | **InDels** | **SNVs** |
| Variants processed | 25868 | 240826 | 21975 | 208505 |
| Variants filtered out | 0 | 0 | 0 | 0 |
| Novel (%) / existing variants (%) | 4515 (17.5) / 21353 (82.5) | 20077 (8.3) / 220749 (91.7) | 3769 (17.2) / 18206 (82.8) | 16920 (8.1) / 191585 (91.9) |
| Overlapped genes | 11837 | 19577 | 10378 | 17631 |
| Overlapped transcripts | 43498 | 67675 | 38280 | 60713 |
| Overlapped regulatory features | 3881 | 14131 | 3407 | 12525 |

# Table S3: Single nucleotide variants (SNVs) found in the LM/Bc strain compared to the C57BL/6J reference.

See attached MS Excel spreadsheet “ Large Supplementary Tables.xlsx”.

# Table S4: Insertions/Deletions (InDels) found in the LM/Bc strain compared to the C57BL/6J reference.

See attached MS Excel spreadsheet “ Large Supplementary Tables.xlsx”.

# Supplementary Figure S1: Sanger Validation *Hnf1b*

Sanger sequencing from 4 LM/Bc mice.

Sanger sequencing of *Hnf1b*. Chromatograms generated from Sanger sequencing of the *Hnf1b* locus in four separate LM/Bc mice over the region of the predicted frameshift variant show that this is likely a false positive call.


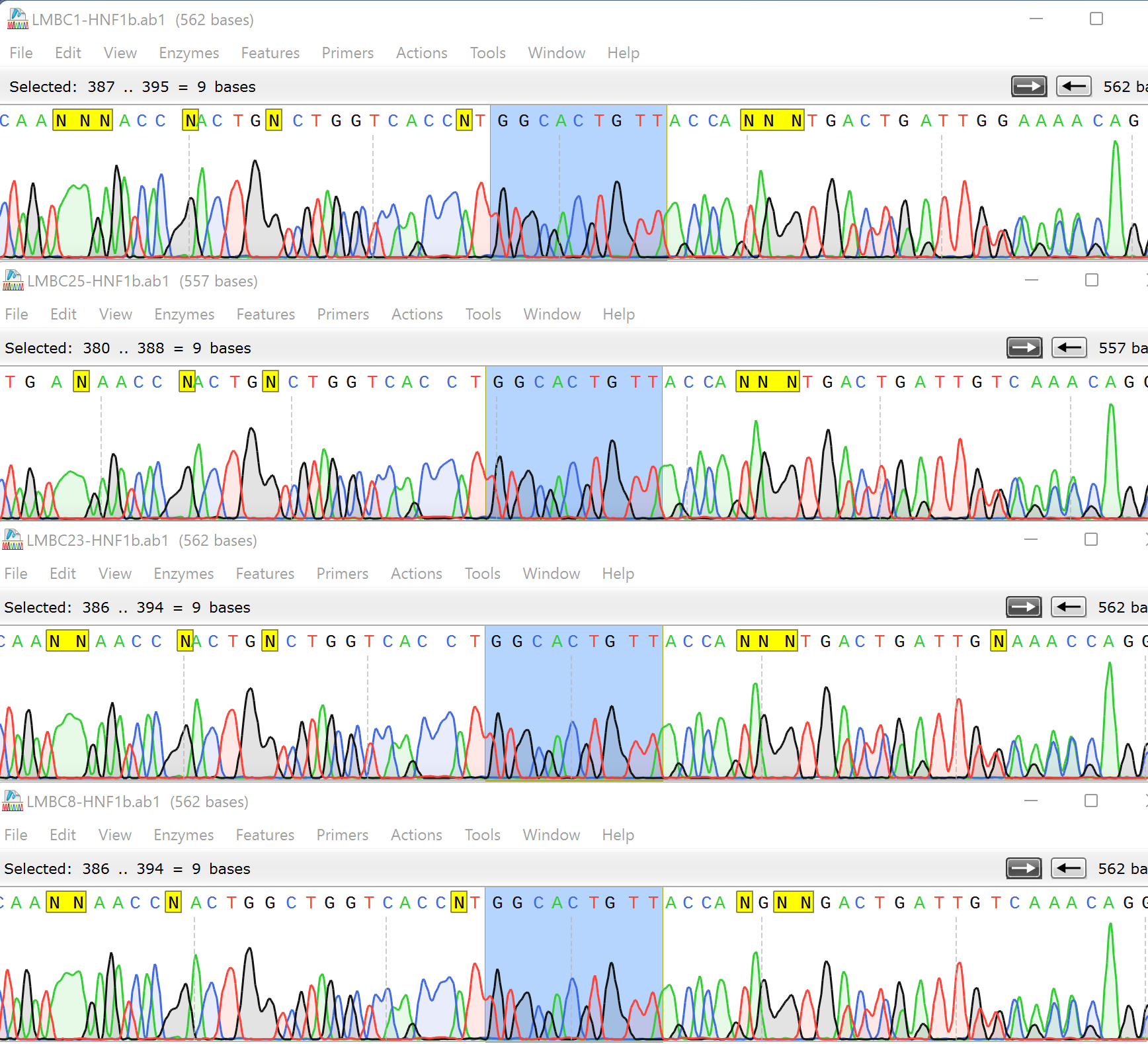


# Supplementary Figure S2. Validation of *Fam111a* variants in mice.

**(A)** Fam111a primers, (B) PCR product predicted using primers (underlined) on mouse tail snip DNA. SNPs predicted (shown in red) in the LM/Bc reverse primer sequence required a unique primer (Fam111a_R2). Only the sequence shown in bold could be clearly resolved from Sanger traces for C-E (not shown).

Sanger-validated **(C)** C57BL/6J, **(D)** SWV, and **(E)** LM/Bc sequences shown with variants from the C57BL/6J reference shown in red.

**A**

*Fam111a primers*

*Forward primer:*

Fam111a_F: TGAGTACACTACATTGAAAGAAGAAGG

Reverse primers:

Fam111a_R (primer used for C57BL/6J and SWV): ATTTCTGAGGGCTGGAAAGG

Fam111a_R2 (primer used for LM/Bc samples): ATTCCTGAGGACTGGAAAGG

**B**

*Fam111a*

Predicted PCR product

GRCm39:chr19:12565002:12565974:1 (shown 5’ – 3’)

TGAGTACACTACATTGAAAGAAGAAGGaaaaaaacttagagcatacatcaaggaaaaaagtgaaaaaaggaagaagaaagcttccttattcaaagtgcataaagaaca**ctttgggaaaatgacaagaaattctactcctgttaaagtggtcaaacatctttcgagggtcagtgactcagttgggttcctatggtggaacaacaatggaaatgcaggctgtgccacctgctttgtttttaaagagttgtacattttgacttgtcagcatgtgatagctagcattgtgggtgaaggcatagattcaagtgagtgggcaaacataattagtcagtgtgtaaaggtgacctttgattatgaagagttactaccaacaggagacaagttttttatggttaaaccttggtttgaaatatctgataaacaccttgactatgctgtcctggaactgaaggaaaatggacaagaagtacctgctgggctgtatcatagaataagacctgtgccacatagtgggttgatttatatcattggccatcctgagggagaaaagaagtctattgattgctgtacagtggtccctcaaagtagtagaagaaaaaaatgtcaggaaaattttcaagcaagagaggaagcaggcttctgtttttctacatcttttatccatatgtacacacaaagaagtttccaggaaatgcttcacaactctgatgtggttacttatgacaccagtttttttggtgggtcttctggatccccagtatttgattctaatggttcattggtggccatgcatgctgctggcatcacttgtacataccaggctggagtttccaatatcattgagtttggttctattatggaatccattga**tgatcatatgaagcaagataaatataaagagtggtataacacaatttctggaaatgttcagaatgtagaaatgctgagcatagATTTCTGAGGGCTGGAAAGG

**C**

Wild-type (C57BL/6J) sequence

>19 dna:chromosome chromosome:GRCm39:19:12565110:12565871:1

CTTTGGGAAAATGACAAGAAATTCTACTCCTGTTAAAGTGGTCAAACATCTTTCGAGGGTCAGTGACTCAGTTGGGTTCCTATGGTGGAACAACAATGGAAATGCAGGCTGTGCCACCTGCTTTGTTTTTAAAGAGTTGTACATTTTGACTTGTCAGCATGTGATAGCTAGCATTGTGGGTGAAGGCATAGATTCAAGTGAGTGGGCAAACATAATTAGTCAGTGTGTAAAGGTGACCTTTGATTATGAAGAGTTACTACCAACAGGAGACAAGTTTTTTATGGTTAAACCTTGGTTTGAAATATCTGATAAACACCTTGACTATGCTGTCCTGGAACTGAAGGAAAATGGACAAGAAGTACCTGCTGGGCTGTATCATAGAATAAGACCTGTGCCACATAGTGGGTTGATTTATATCATTGGCCATCCTGAGGGAGAAAAGAAGTCTATTGATTGCTGTACAGTGGTCCCTCAAAGTAGTAGAAGAAAAAAATGTCAGGAAAATTTTCAAGCAAGAGAGGAAGCAGGCTTCTGTTTTTCTACATCTTTTATCCATATGTACACACAAAGAAGTTTCCAGGAAATGCTTCACAACTCTGATGTGGTTACTTATGACACCAGTTTTTTTGGTGGGTCTTCTGGATCCCCAGTATTTGATTCTAATGGTTCATTGGTGGCCATGCATGCTGCTGGCATCACTTGTACATACCAGGCTGGAGTTTCCAATATCATTGAGTTTGGTTCTATTATGGAATCCATTGA

**D**

>SWV2086_sequence (same as C57BL/6J)

CTTTGGGAAAATGACAAGAAATTCTACTCCTGTTAAAGTGGTCAAACATCTTTCGAGGGTCAGTGACTCAGTTGGGTTCCTATGGTGGAACAACAATGGAAATGCAGGCTGTGCCACCTGCTTTGTTTTTAAAGAGTTGTACATTTTGACTTGTCAGCATGTGATAGCTAGCATTGTGGGTGAAGGCATAGATTCAAGTGAGTGGGCAAACATAATTAGTCAGTGTGTAAAGGTGACCTTTGATTATGAAGAGTTACTACCAACAGGAGACAAGTTTTTTATGGTTAAACCTTGGTTTGAAATATCTGATAAACACCTTGACTATGCTGTCCTGGAACTGAAGGAAAATGGACAAGAAGTACCTGCTGGGCTGTATCATAGAATAAGACCTGTGCCACATAGTGGGTTGATTTATATCATTGGCCATCCTGAGGGAGAAAAGAAGTCTATTGATTGCTGTACAGTGGTCCCTCAAAGTAGTAGAAGAAAAAAATGTCAGGAAAATTTTCAAGCAAGAGAGGAAGCAGGCTTCTGTTTTTCTACATCTTTTATCCATATGTACACACAAAGAAGTTTCCAGGAAATGCTTCACAACTCTGATGTGGTTACTTATGACACCAGTTTTTTTGGTGGGTCTTCTGGATCCCCAGTATTTGATTCTAATGGTTCATTGGTGGCCATGCATGCTGCTGGCATCACTTGTACATACCAGGCTGGAGTTTCCAATATCATTGAGTTTGGTTCTATTATGGAATCCATTGA

**E**

>LMBc3103_sequence (SNPs in red)

**G**TTTGGGAAAATGA**A**AAG**T**AATTCTACTCCTGTTA**C**AGTG**CA**CGAAAATCTTTCGAGGGTCAGTG**C**CTCAGTTGGGT**A**CCTATGGTGG**G**ACAACAATGGAAATGCAGGCTGTGCCACCTGCTTTGTTTTTAAAGAGTTGTACATTTTGACTTGTCAGCATGTGATAGCTAGCATTGTGGGTGAAGGCATAGATTCAAGTGAGTGGGCAA**G**CATAATTAGTCAGTGTGTAAAGGTGACCTTTGATTA**CA**AAGA**T**TT**T**C**C**AC**T**AACA**AA**AGACAAGTTTTTTATGGTTAAACCTTGGTTTGA**G**ATATC**C**GATAAA**G**ACCTTGACTATGCTGTCCTGGAACTGAAGGAAAATGGACAAGAAGTACCTGCTGGGCTGTAT**A**AT**G**GAATA**G**GACCTGTGCCAC**T**T**G**GTGGGTTGATTTATATCATTGGCCATCCTGAGGGAG**G**AAAGAAGTCTA**G**TGAT**G**GCTGTACAGTGGTCCCTCAA**G**GTAGTAGAAGAAAAAAATGTCAGGAAAATTTTCAAGCAAGAGAGGAAGCAGGCT**G**CTGTTTTTCTACATCTTTTATCCATATGTACACACAAAGAAGTTTCCAGGAAATGCTTCACAACTCTGATGTG**A**TTACTTATGACACCAGTTTTTTTGGTGGGTCTTCTGGATCCCCAGTATTTGATTCTAATGGTTCATTGGTGGCCATGCATGCTGCTGGCATCACTTGTACATACCAGGCTGGAGTTTCCA**G**TATCATTG**N**GTTTGGTTCTA**C**TATGGAATCCATT**CT**
